# Developing a Detailed Chemical Kinetic Model for Combustion of *Iso*-Cetane Based on Ignition and Oxidation

**DOI:** 10.3390/molecules31091403

**Published:** 2026-04-23

**Authors:** Pan Chen, Yijun Heng, Bohui Zhao, Neng Zhu, Junjie Liang, Gesheng Li

**Affiliations:** 1 School of Naval Architecture, Ocean and Energy Power Engineering, Wuhan University of Technology, Wuhan 430063, China; chenpan123@whut.edu.cn (P.C.); bohuizhao@whut.edu.cn (B.Z.); gsliwh@gmail.com (G.L.); 2 Ninghai College, Zhejiang Business Technology Institute, Ningbo 315012, China; yijunheng@zbti.edu.cn; 3 School of Automotive and Transportation Engineering, Wuhan University of Science and Technology, Wuhan 430081, China

**Keywords:** *iso*-cetane, detailed kinetic model, ignition delay times, chemical kinetic analysis

## Abstract

*Iso*-cetane serves as an ideal component representing branched-chain alkanes in surrogate fuels for diesel. However, the predictive accuracy of existing detailed chemical kinetic models for *iso*-cetane requires improvement. In this study, focusing on the reaction processes of *iso*-cetane and its key intermediates, we first updated the thermodynamic data of *iso*-cetane and some of its intermediates, systematically analyzed the effects of various reactions on ignition delay time (IDT), and made targeted modifications to the relevant reaction rate constants. The reaction types involved include fuel cracking reactions of *iso*-cetane, hydrogen abstraction reactions, cracking reactions of fuel radicals, as well as the oxidation of fuel radicals, isomerization of alkylperoxy radicals ($R{\dot{O}}_{2}$), concerted elimination reactions, formation of cyclic ethers, and the formation and decomposition of ketohydroperoxides (KHP). Additionally, reactions related to the formation and consumption of *p*-alkyl-dihydroperoxides ($\dot{P}{\left(\mathrm{OOH}\right)}_{2}$) were supplemented. Based on the above work, we developed a detailed chemical kinetic model for *iso*-cetane, comprising 4541 species and 18,359 elementary reactions. Through systematic validation against experimental data on ignition delay time and concentration variations of key species during oxidation, the improved predictive performance of the proposed model was demonstrated. Furthermore, using sensitivity analysis and reaction pathway analysis for the ignition process, we revealed that the formation of the low-temperature negative temperature coefficient (NTC) region for *iso*-cetane is intrinsically associated with the competition between chain-branching and chain-propagating pathways.

## 1. Introduction

Currently, diesel fuel continues to play a significant role in transportation energy, serving not only as fuel for ships, agricultural machinery, heavy-duty vehicles, and other applications, but also as pilot fuel for engines utilizing low-carbon and zero-carbon fuels such as methanol and ammonia. Due to the extreme complexity of actual diesel fuel, which contains hundreds or even thousands of species, researchers typically employ surrogate fuel to simulate real diesel for study purposes [1]. In diesel surrogate fuel, *iso*-cetane (2,2,4,4,6,8,8-heptamethylnonane, $i\text{-}C_{16}H_{34}$) is often selected as the representative branched-chain hydrocarbon component. *Iso*-cetane serves as a high-branched alkane reference compound for determining cetane ratings. Its carbon atom count falls within the mid-range of diesel species ($C_{10}–C_{22}$), effectively balancing molecular weights [2].

Numerical simulation methods coupling fluid dynamics and fuel chemical kinetic models are crucial tools for studying in-cylinder combustion processes, aiding our investigation of fuel combustion characteristics. Therefore, a chemical kinetic model with excellent predictive performance is essential. Currently, researchers have conducted experimental and mechanistic simulation studies on *iso*-cetane. Experiments primarily involve measuring ignition delay times using a shock tube (ST) and a rapid compression machine (RCM), alongside measuring species concentration profiles in a jet-stirred reactor (JSR) [3,4,5,6]. Oehlschlaeger et al. [3] pioneered gas-phase autoignition experiments for *iso*-cetane in a shock tube and measured ignition delay times for *iso*-cetane/air mixtures across temperatures (879–1347 K), pressures (8–47 atm), and equivalence ratios (0.5, 1, and 1.5). Yu et al. [4] conducted low-temperature autoignition experiments on *iso*-cetane using a rapid compression machine, measured ignition delay times for *iso*-cetane/air mixtures at temperatures of 620–880 K, pressures of 10–20 bar, and equivalence ratios of 0.5–2.0. Raza et al. [5] measured the ignition delay times of *iso*-cetane/air mixtures at temperatures ranging from 838 K to 1617 K, pressures from 10 to 20 bar, and equivalence ratios from 0.5 to 2.0. Dagaut et al. [6] conducted oxidation experiments on *iso*-cetane using a jet-stirred reactor within the temperature range of 770–1070 K.

Regarding the mechanisms of *iso*-cetane, Oehlschlaeger et al. [3], Yu et al. [4], and Raza et al. [5] proposed detailed kinetic mechanisms. Additionally, Ranzi [7], Zhu et al. [8], and Chang et al. [9] investigated the skeletal mechanism and simplified mechanism of *iso*-cetane. Oehlschlaeger et al. [3] constructed a detailed chemical kinetic model for *iso*-cetane comprising 1114 species and 4469 elementary reactions, based on the *iso*-octane mechanism [10] and straight-chain alkane mechanism [11]. They validated experimental ignition delay time data under high-temperature conditions, with predicted values showing good agreement with experimental results. However, this model exhibits poor predictive accuracy for ignition delay times at low temperatures and lacks concerted elimination reactions ($R{\dot{O}}_{2}$ = alkene + $H{\dot{O}}_{2}$, ${\dot{O}}_{2}\mathrm{QOOH}$ = EROOH + $H{\dot{O}}_{2}$). Yu et al. [4] updated the Oehlschlaeger mechanism [3] based on the latest *iso*-octane mechanism [12], upgraded thermodynamic data for certain species, replaced the $C_{0}–C_{4}$ core mechanism with AramcoMech 2.0 [13], and added new isomerization and oxygenation reaction pathways. The updated mechanism incorporated 2458 species and 9685 elementary reactions. The updated mechanism showed improved predictive performance in the medium-to-high temperature range. Validation against experimental data on ignition delay times revealed that the model underestimated ignition delay times under low-to-medium temperature conditions. It misjudged the temperature range for the ignition delay NTC of *iso*-cetane, with model predictions being approximately 80 K higher than experimental results. Building upon the work of Yu et al. [4], Raza et al. [5] updated the mechanism by introducing new reaction categories: alkyl peroxy radical isomerization ($R{\dot{O}}_{2}$ = $\dot{Q}\mathrm{OOH}$), concerted elimination reactions ($R{\dot{O}}_{2}$ = alkene + $H{\dot{O}}_{2}$), cyclic ether formation ($\dot{Q}\mathrm{OOH}$= cyclic ether + $\dot{O}H$), and ketohydroperoxide formation (${\dot{O}}_{2}\mathrm{QOOH}$ = KHP + $\dot{O}H$). However, the reaction ${\dot{O}}_{2}\mathrm{QOOH}$ = EROOH + $H{\dot{O}}_{2}$ remained absent. The updated mechanism comprised 2465 species and 10,348 elementary reaction steps. Verification against ignition delay time data revealed improved predictive performance for the NTC region and higher temperature region in the updated mechanism, but deviations persisted in predicting ignition delay times at lower temperatures. Ranzi [7] developed a framework mechanism incorporating the *iso*-cetane mechanism to generate ignition delay time data for mixed fuels in heated shock tubes. Based on the comprehensive mechanism for fossil and bio-derived fuels developed by Ranzi [7], Zhu et al. [8] simplified the *iso*-cetane mechanism by removing redundant reactions and species unrelated to *iso*-cetane reactions. The final simplified mechanism comprises 83 species and 299 elementary reactions. Chang et al. [9] proposed a novel approach to reduce large-scale reaction kinetics mechanisms by performing global sensitivity analysis on reaction classes/sub-mechanisms within the mechanism. This simplified the Oehlschlaeger mechanism [3] into a new *iso*-cetane mechanism comprising 56 species and 131 reaction steps. The detailed mechanisms developed in the above studies accurately predict ignition delay times under high-temperature conditions but exhibit deviations in predicting ignition delay times under medium-to-low-temperature conditions. Furthermore, the reaction pathways are not comprehensive, lacking certain reaction categories.

Mechanisms for macromolecular hydrocarbons are typically constructed using a layered approach, comprising core mechanisms at the small-molecule level ($C_{0}–C_{5}$) and sub-mechanisms at the fuel level with varying carbon numbers. The core mechanisms of the detailed kinetic mechanism for *iso*-cetane were developed in disparate studies, which hinder the development of diesel surrogate fuel and need to be updated. Zhang et al. [14] systematically investigated the oxidative combustion behavior of five hexane isomers, developed a $C_{6}$ kinetic model incorporating different alkane structural isomers based on experimental data, and derived reaction rate rules. However, when Heng et al. [15] developed the $C_{8}$ mechanism based on these rules, the predictions exhibited certain deviations. Therefore, whether these rate rules can be extended to the $C_{16}$ level remains to be verified.

Therefore, this work first investigated the ability to extend the aforementioned $C_{6}$ rate rules to the $C_{16}$ system. A detailed chemical kinetic model for *iso*-cetane was developed by updating thermodynamic data and the core mechanism, supplementing missing reaction classes, and updating reaction rate constants. It was used to analyze the impact of reaction rate constants for key reactions within the kinetic mechanism on ignition delay times. The predictive accuracy of the mechanism was validated using experimental data on ignition delay times and concentrations of key species. Additionally, kinetic analyses were performed on the basis of the developed mechanism.

## 2. Results and Discussion

### 2.1. Validation of Chemical Kinetic Model

To validate the predictive accuracy of the kinetic model developed in the present work, it was compared with models developed by Oehlschlaeger et al. [3] and Raza et al. [5], along with corresponding experimental data. The experimental data include ignition delay times and concentrations of key species during oxidation. Ignition delay time data are sourced from Raza et al. [5] and Yu et al. [4], while concentrations of key oxidation species are sourced from Dagaut et al. [6].

**Figure 1:**
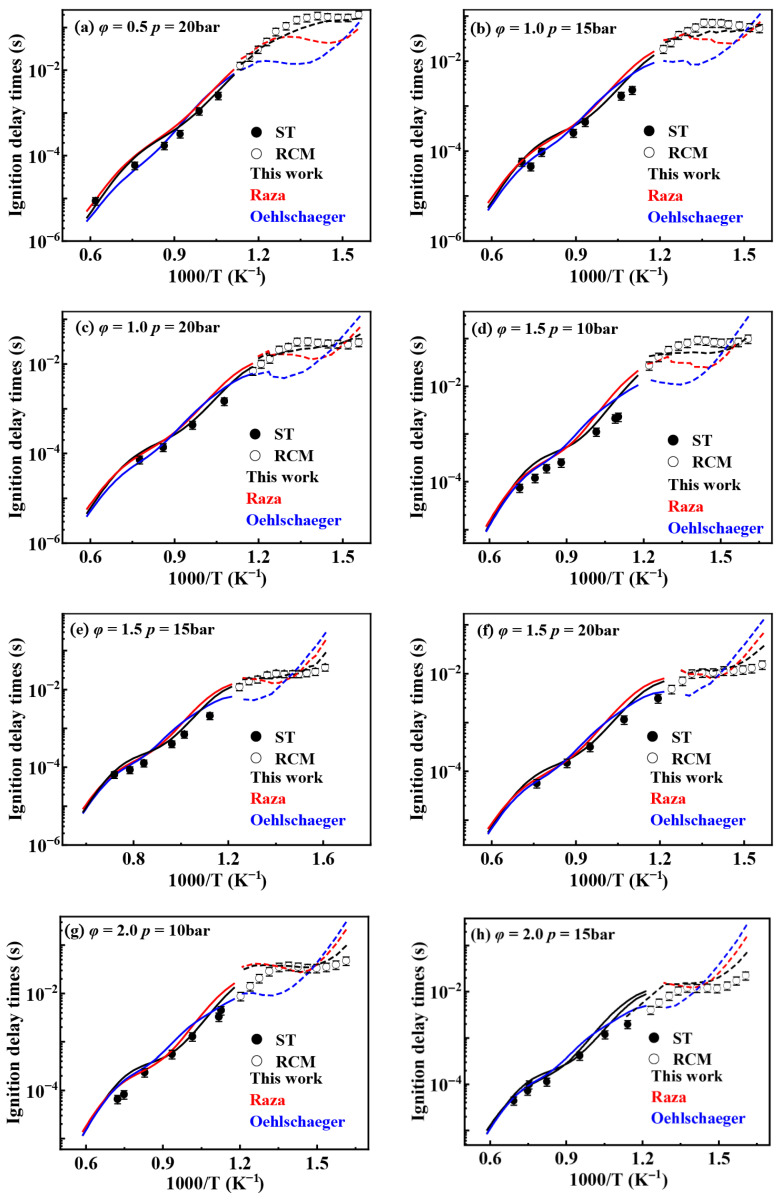
Comparisons of simulated and experimental ignition delay times for *iso*-cetane (solid symbols: experimental data from shock tube [5]; open symbols: experimental data from rapid compression machine [4]; solid lines: constant volume simulation; dash lines: variable volume simulation; black lines: the present mechanism; red lines: Raza mechanism [5]; blue lines: Oehlschlaeger mechanism [3]). (**a**) *φ* = 0.5, *p* = 20 bar; (**b**) *φ* = 1.0, *p* = 15 bar; (**c**) *φ* = 1.0, *p* = 20 bar; (**d**) *φ* = 1.5, *p* = 10 bar; (**e**) *φ* = 1.5, *p* = 15 bar; (**f**) *φ* = 1.5, *p* = 20 bar; (**g**) *φ* = 2.0, *p* = 10 bar; (**h**) *φ* = 2.0, *p* = 15 bar.

Figure 1 shows the comparison between simulated and experimental ignition delay times for different kinetic models under pressures of 10, 15, and 20 bar and equivalence ratios of 0.5, 1.0, 1.5, and 2.0. The results indicate that all mechanisms exhibit good agreement between simulated and experimental ignition delay times under high-temperature conditions (>900 K). However, in the low-temperature region, particularly the NTC region, the ignition delay times from the Oehlschlaeger and Raza kinetic models deviate from experimental values. Furthermore, these kinetic models misjudge the temperature range of the NTC region, predicting its onset to occur several tens of kelvins higher than experimentally observed. The model developed in the present work effectively predicts the extent of the NTC region. It also demonstrates good agreement between simulated and experimental ignition delay times at low temperatures under most operating conditions, with deviations occurring only in certain scenarios. Even when deviations exist, this model exhibits the smallest discrepancies among the kinetic models.

**Figure 2:**
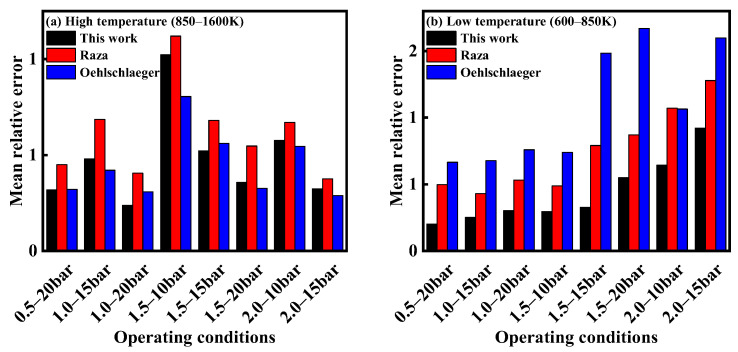
The mean relative errors between the ignition delay times predicted by each mechanism and the experimental values for *iso*-cetane/air mixtures under different operating conditions. (**a**) *T* = 850–1600 K; (**b**) *T* = 600–850 K.

To further evaluate the predictive performance of different kinetic models, this study calculated the mean relative error (MRE) between the ignition delay times of *iso*-cetane/air mixtures predicted by each kinetic model and the experimental values under various operating conditions. The calculation formula is shown as Equation (1):

(1) $$\mathrm{MRE}=\frac{1}{n}\sum_{i=1}^{n}\left|\frac{y_{i}-{\hat{y}}_{i}}{y_{i}}\right|$$

where *n* means the total number of the conditions; ${\hat{y}}_{i}$ and $y_{i}$ indicate the *i*-th predicted value from the kinetic model and the experimental value, respectively, under the *i*-th condition. The experimental data under high-temperature conditions were taken from the work of Raza et al. [5], while those under low-temperature conditions were taken from the study by Yu et al. [4]. The comparison results are shown in Figure 2. From Figure 2a, it can be seen that under high-temperature conditions (850–1600 K), the mean relative errors of the kinetic model constructed in this study are similar to those of the kinetic model constructed by Oehlschlaeger et al. [3], and under most conditions, the mean relative errors are below 50%. Only at an equivalence ratio of 1.5 and a pressure of 10 bar does the mean relative error become larger, reaching 100%. The mean relative errors of the kinetic model constructed by Raza et al. [5] are greater than those of the other two kinetic models under all conditions, and under most conditions, the mean relative errors exceed 50%, with a maximum mean relative error of over 110%. From Figure 2b, it can be seen that under low-temperature conditions (600–850 K), the kinetic model constructed in this study exhibits mean relative errors below 50% under most conditions, and under all conditions, the mean relative errors are smaller than those of the other two kinetic models. The mean relative errors of the kinetic model constructed by Oehlschlaeger et al. [3] are all above 50%, with a maximum mean relative error of over 150%. The mean relative errors of the kinetic model constructed by Raza et al. [5] increase continuously with increasing equivalence ratio and pressure, reaching a maximum mean relative error of over 110%. In summary, the kinetic model constructed in this study shows predictive accuracy under high-temperature conditions comparable to that of existing kinetic models (Oehlschlaeger et al. [3], Raza et al. [5]), while its predictive accuracy under low-temperature conditions is significantly improved compared to the existing models.

**Figure 3:**
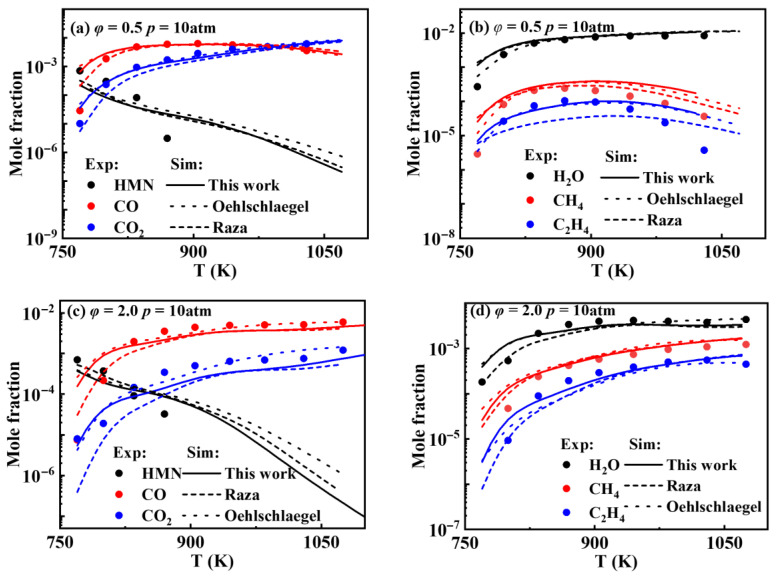
Comparisons between the simulated and experimental values for concentrations of key species during *iso*-cetane oxidation process. Symbols and lines denote experimental data from the study of Dagaut et al. [6] and modeled values, respectively (solid lines: the present model; dash lines: Raza model [5]; short dash lines: Oehlschlaeger model [3]). (**a**) *φ* = 0.5, *p* = 10 atm—concentration profiles of HMN, CO, and $CO_{2}$; (**b**) *φ* = 0.5, *p* = 10 atm—concentration profiles of $H_{2}O$, $CH_{4}$ and $C_{2}H_{4}$; (**c**) *φ* = 2.0, *p* = 10 atm—concentration profiles of HMN, CO, and $CO_{2}$; (**d**) *φ* = 2.0, *p* = 10 atm—concentration profiles of $H_{2}O$, $CH_{4}$ and $C_{2}H_{4}$.

Figure 3 presents the comparison between the simulated and experimental values for concentrations of key species during the *iso*-cetane oxidation process. It is evident that all three detailed models exhibit deviations in predicting *iso*-cetane concentration changes beyond 850 K, underestimating the reaction rate constants of *iso*-cetane and resulting in simulated values exceeding experimental values. For CO, $CO_{2}$, and $H_{2}O$, the simulated values of concentrations from all three models agree well with the experimental ones initially, but the agreement deteriorates progressively as the equivalence ratio increases. Regarding $CH_{4}$ and $C_{2}H_{4}$, the three models reproduce well experimental data below 900 K. Above 900 K, simulated values exceed experimental values, and the agreement improves progressively with the increasing equivalence ratio.

### 2.2. Reaction Pathway and Sensitivity Analyses

**Figure 4:**
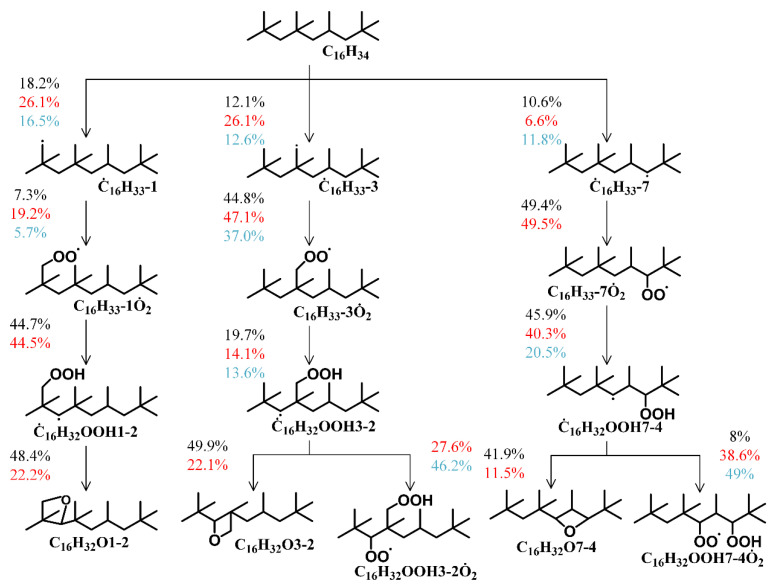
Comparison of *iso*-cetane reaction pathways among three reaction models at *T* = 700 K, *φ* = 1.0 and *p* = 15 bar (black numbers: the present work model; red numbers: Raza model [5]; blue numbers: Oehlschlaeger model [3]). All dots in the figure represent unpaired electrons.

Figure 4 displays the reaction pathways for *iso*-cetane oxidation from the present work and from the models of Raza et al. [5] and Oehlschlaeger et al. [3], under conditions of *T* = 700 K, *φ* = 1.0, and *p* = 15 bar. The black, red, and blue lines represent the reaction pathways for the mechanisms in the present work, Raza et al. [5] and Oehlschlaeger et al. [3], respectively. It can be observed that in the reaction pathways of the Raza and Oehlschlaeger models, the primary pathway for the hydroperoxyalkyl radical ($\dot{Q}OOH$) involves oxygen addition to form peroxyhydroperoxyalkyl (${\dot{O}}_{2}QOOH$), followed by ketohydroperoxide formation and decomposition. This pathway belongs to the low-temperature chain-branching pathway, promoting ignition and reducing ignition delay times, leading the model to underestimate ignition delay under low-temperature conditions. In contrast, the primary pathway for hydroperoxyalkyl radical groups in the present work involves forming cyclic ethers, followed by their cleavage. This pathway belongs to the low-temperature chain-propagation pathway, competing with the low-temperature chain-branching pathway to inhibit ignition and increase ignition delay times. Simultaneously, the competition between the low-temperature chain-branching pathway and the low-temperature chain-propagation pathway is also a key factor contributing to the NTC region of *iso*-cetane at low temperatures.

**Figure 5:**
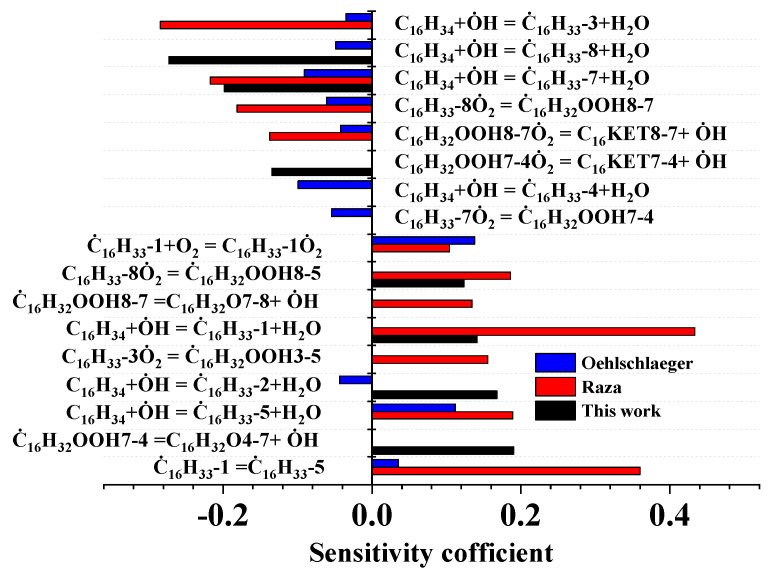
Sensitivity of *iso*-cetane IDT to elementary reactions at *φ* = 1.0, *p* = 30 atm, and *T* = 700 K.

To identify the key reactions controlling *iso*-cetane ignition under the same conditions as those used in the reaction pathway analysis, a sensitivity analysis was conducted in this study, with the sensitivity coefficient defined as shown in Equation (2) [16]. This formulation eliminates dimensional effects through normalization, making the relative contributions of different reaction rate constants to the ignition delay time directly comparable. The results are shown in Figure 5.

(2) $$S=\frac{\tau (2.0k_{i})-\tau (0.5k_{i})}{1.5\tau (k_{i})}$$

Here, *S* denotes the sensitivity coefficient, $k_{i}$ represents the rate constant of the *i*-th elementary reaction, and $\tau (k_{i})$ indicates the ignition delay time under the rate constant of the *i*-th elementary reaction, $\tau (2.0k_{i})$ indicates the ignition delay time calculated using twice the original rate constant, and $\tau (0.5k_{i})$ indicates the ignition delay time calculated using half the original rate constant. A positive sensitivity coefficient indicates that increasing the reaction rate of reaction *i* prolongs the ignition delay time, meaning that the reaction inhibits ignition. A negative sensitivity coefficient indicates that increasing the reaction rate of reaction *i* shortens the ignition delay time, meaning that the reaction promotes ignition. As shown in Figure 5, most reactions exhibit identical effects on fuel ignition across different mechanisms, with only a few reactions showing discrepancies. For instance, the reaction $C_{16}H_{34}$ + $\dot{O}H$ = ${\dot{C}}_{16}H_{33}\text{-}2$ + $H_{2}O$ promotes ignition in the Oehlschlaeger model but inhibits ignition in the present work. In all three models, hydrogen extraction reactions between fuel molecules and $\dot{O}H$ significantly affect ignition delay times, though the specific reactions involved differ. In the model proposed in the present work, the reactions $C_{16}H_{34}$ + $\dot{O}H$ = ${\dot{C}}_{16}H_{33}\text{-}1$ + $H_{2}O$, $C_{16}H_{34}$ + $\dot{O}H$ = ${\dot{C}}_{16}H_{33}\text{-}2$ + $H_{2}O$, $C_{16}H_{34}$ + $\dot{O}H$ = ${\dot{C}}_{16}H_{33}\text{-}7$ + $H_{2}O$, and $C_{16}H_{34}$ + $\dot{O}H$ = ${\dot{C}}_{16}H_{33}\text{-}8$ + $H_{2}O$ exert substantial influence. In the model of Raza, reactions $C_{16}H_{34}$ + $\dot{O}H$ = ${\dot{C}}_{16}H_{33}\text{-}1$ + $H_{2}O$, $C_{16}H_{34}$ + $\dot{O}H$ = ${\dot{C}}_{16}H_{33}\text{-}3$ + $H_{2}O$, $C_{16}H_{34}$ + $\dot{O}H$ = ${\dot{C}}_{16}H_{33}\text{-}5$ + $H_{2}O$, and $C_{16}H_{34}$ + $\dot{O}H$ = ${\dot{C}}_{16}H_{33}\text{-}7$ + $H_{2}O$ exert greater influence. In the model of Oehlschlaeger, the reactions $C_{16}H_{34}$ + $\dot{O}H$ = ${\dot{C}}_{16}H_{33}\text{-}1$ + $H_{2}O$, $C_{16}H_{34}$ + $\dot{O}H$ = ${\dot{C}}_{16}H_{33}\text{-}4$ + $H_{2}O$, $C_{16}H_{34}$ + $\dot{O}H$ = ${\dot{C}}_{16}H_{33}\text{-}5$ + $H_{2}O$, and $C_{16}H_{34}$ + $\dot{O}H$ = ${\dot{C}}_{16}H_{33}\text{-}7$ + $H_{2}O$ exert significant influence. By examining the reaction pathways depicted above, we observe that reactions occurring along chain-branching pathways, such as $C_{16}H_{32}OOH7\text{-}4{\dot{O}}_{2}$ = $C_{16}\mathrm{KET}7\text{-}4$ + $\dot{O}H$ and $C_{16}H_{32}OOH8\text{-}7{\dot{O}}_{2}$ = $C_{16}\mathrm{KET}8\text{-}7$ + $\dot{O}H$, all promote ignition, while reactions occurring along the chain-propagation pathway, such as ${\dot{C}}_{16}H_{32}OOH7\text{-}4$ = $C_{16}H_{32}O4\text{-}7$ + $\dot{O}H$ and ${\dot{C}}_{16}H_{32}OOH8\text{-}7$ = $C_{16}H_{32}O7\text{-}8$ + $\dot{O}H$, inhibit ignition.

## 3. Construction of Chemical Kinetic Model for *Iso*-Cetane

### 3.1. Overview of Development of the Chemical Kinetic Model

The construction of the kinetic model for *iso*-cetane primarily encompassed two core components: thermodynamic data and chemical kinetic mechanisms. The thermodynamic data part includes fundamental thermophysical parameters of various species, such as enthalpy, entropy, and specific heat capacity [17]. In this study, the group additivity method was employed to update the thermodynamic data on *iso*-cetane and its related components. The core principle of this method lies in decomposing complex molecules into combinations of functional groups and chemical bonds, and subsequently estimating the thermodynamic properties of unknown species based on the group additivity values (GAVs) of known structures.

The chemical kinetic mechanism consists of a reaction network composed of various species and their corresponding elementary reactions (including rate constants). For large hydrocarbon fuels, mechanism development generally adopts a hierarchical approach [18], which first selects a core reaction mechanism covering small molecules ($C_{0}–C_{5}$) as a foundational framework, and then progressively incorporates a high-carbon-number sub-mechanism for the specific fuel to form a complete reaction description system. In this study, NUIG-Mech1.3 [19,20] was adopted as the core mechanism. The fuel sub-mechanism referenced the framework of existing detailed kinetic mechanisms for *iso*-cetane and alkane oxidation mechanisms [21], while the reaction rate constants were uniformly assigned according to the alkane rate rules proposed by Zhang et al. [14]. The specific reaction classes included in this mechanism are shown in Table 1. To further improve the predictive accuracy of the model, the kinetic parameters of certain key reactions were calibrated and optimized, with all adjustments made within the uncertainty ranges of the corresponding studies.

All kinetic simulations were performed using the CHEMKIN-PRO 15131 software platform. The simulation of ignition delay time was conducted using a closed homogeneous reactor under adiabatic and constant-pressure conditions, with the ignition delay time taken as the time at which the first derivative of the $\dot{O}H$ mole fraction with respect to time reaches its maximum value. The simulation of key species concentration profiles during the oxidation process was carried out using a perfectly stirred reactor model.

### 3.2. Thermochemistry

The present work calculated thermodynamic data for *iso*-cetane-related species using the THERM code, based on the group additivity method proposed by Benson et al. [22,23], and employed optimized GAVs from Ghosh et al. [24,25]. These GAVs were optimized based on high-precision quantum chemical calculations by Elliott et al. [26,27]. This study was the first to account for the interaction between alkyl groups and -OOH groups, which increased the formation enthalpies of alkyl peroxy radicals ($R{\dot{O}}_{2}$), hydroperoxyalkyl radicals ($\dot{Q}OOH$), and peroxyhydroperoxyalkyl radicals (${\dot{O}}_{2}QOOH$). This interaction significantly impacted the ignition delay times of the model in the low-temperature region.

**Figure 6:**
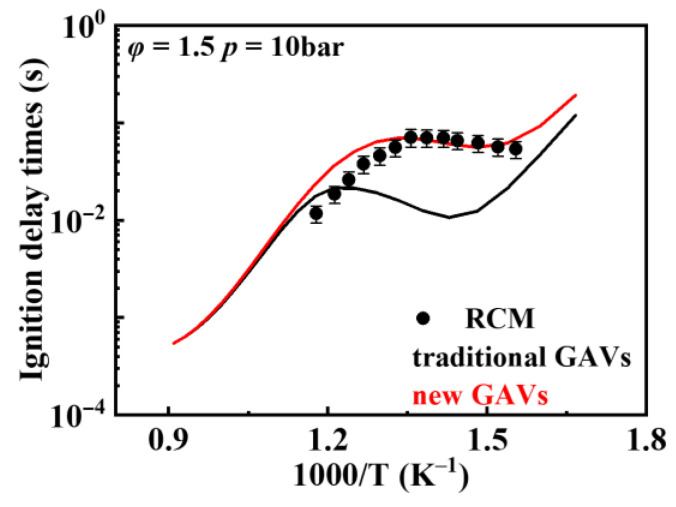
Effect of thermodynamic data using new and traditional GAVs on simulated ignition delay times for *iso*-cetane (symbol: experimental data from rapid compression machine [4]; black line: mechanism using the traditional GAVs; red line: mechanism using the new GAVs).

Figure 6 compares the effect of thermodynamic data on the ignition delay times of the *iso*-cetane kinetic model when using new and traditional GAVs. “Traditional GAVs” refer to the group additivity values used in the *iso*-cetane kinetic model constructed by Raza et al. [5], which were mainly based on parameterization results from smaller alkanes (e.g., *iso*-octane). “New GAVs” refer to the group additivity values updated by Ghosh et al. [24,25]. The figure shows that updating the thermodynamic data significantly impacts the ignition delay times under low-temperature conditions (<900 K), but has a smaller effect on ignition delay times under medium-to-high-temperature conditions, particularly in the NTC region. The new GAVs effectively reduce the reactivity in the NTC region, leading to increased ignition delay times in this area and improved predictive performance.

To identify species with a significant influence on mechanistic performance, the present work investigated the sensitivity of ignition delay times to the thermodynamic data of species. The Gibbs free energy *G* of each species in the mechanism is calculated based on its thermodynamic data using the following relationship (3):

(3) G=H−TS

where *T* represents temperature, and *G*, *H*, and *S* denote the Gibbs free energy, enthalpy, and entropy of the species at temperature *T*, respectively. The enthalpy and entropy of the species are calculated from its thermodynamic data. The specific procedure is that for species *i*, the Gibbs free energy is increased by 1 kcal·mol^−1^, and the corresponding simulated ignition delay times (IDT*_i_*) are calculated. Using the initial simulated ignition delay times (IDT_0_) as the baseline, the sensitivity coefficient *S_i_* [15] for the thermodynamic data of species *i* is calculated via Equation (4):

(4) $$S_{i}=\frac{\mathrm{ID}T_{i}-\mathrm{ID}T_{0}}{\mathrm{ID}T_{0}}$$

**Figure 7:**
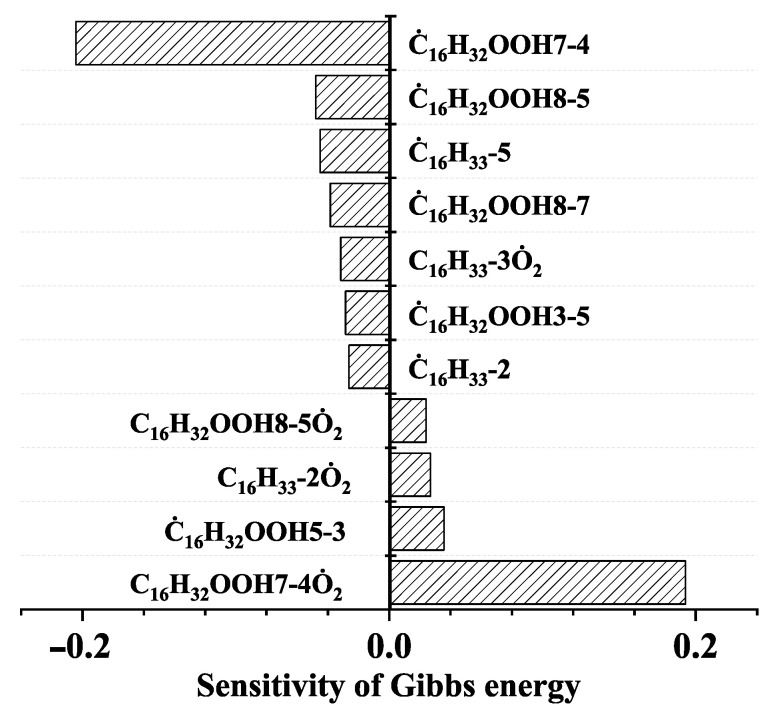
Sensitivity coefficients for Gibbs free energy of species at *T* = 700 K, *φ* = 1.0 and *p* = 15 bar.

**Figure 8:**
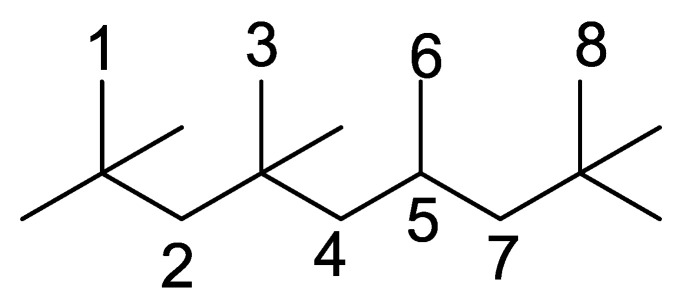
Carbon chain numbering of *iso*-cetane. The numbers (1, 2, 3, …) denote the carbon atom numbering scheme used to identify branching points and terminal positions.

**Figure 9:**
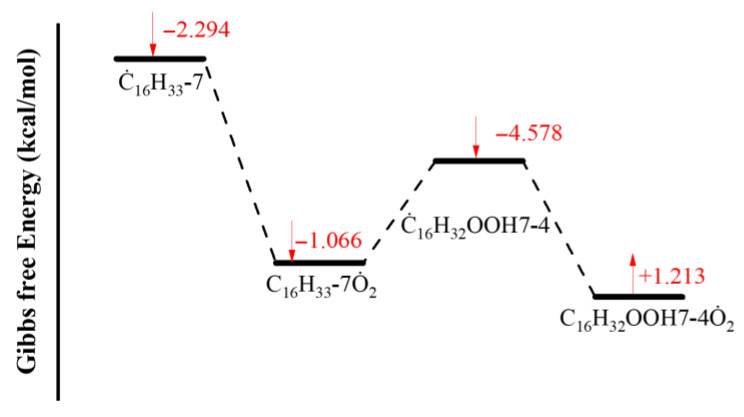
Changes in Gibbs free energy at 298.15 K after updating GAVs. The arrows indicate an increase or decrease in the free energy of the species.

To further investigate the influence of thermodynamic data from different species on the ignition delay times of *iso*-cetane, the present work calculated the sensitivity coefficient of ignition delay times to the thermodynamic data of each substance within the NTC region (700 K). The results are shown in Figure 7, and the specific structures of species involved are listed in Table 2, namely the molecular structures of the major species involved in the low-temperature oxidation of *iso*-cetane. The table presents the chemical structural formulas and the naming rules based on carbon atom position numbering for each species. This table serves as a cross-reference index for the naming rules throughout the manuscript, improving the readability and professionalism of the paper. The numbers (e.g., -4, -7) in the naming of species in Table 2 represent the position numbers of substituents on the carbon chain in the corresponding chemical structures. For example, “${\dot{C}}_{16}H_{33}\text{-}7$” means that *iso*-cetane has lost a hydrogen atom at position 7; “$C_{16}H_{33}\text{-}7{\dot{O}}_{2}$” indicates that there is a peroxy group (-OO) at position 7; and “${\dot{C}}_{16}H_{32}OOH7\text{-}4$” means that there is a hydroperoxy group (-OOH) at position 7 and a hydrogen atom is missing at position 4. Figure 8 presents the carbon chain skeleton of *iso*-cetane and its numbering scheme, providing an intuitive structural reference for the numbers in the species structures listed in Table 2. Figure 7 indicates that the thermodynamic data for species such as ${\dot{C}}_{16}H_{32}OOH7\text{-}4$ and $C_{16}H_{32}OOH7\text{-}4{\dot{O}}_{2}$ exert the greatest influence on the simulated ignition delay times of *iso*-cetane within the NTC region. These species originate from the following reaction pathway: ${\dot{C}}_{16}H_{33}\text{-}7$ ⇌ $C_{16}H_{33}\text{-}7{\dot{O}}_{2}$ ⇌ ${\dot{C}}_{16}H_{32}OOH7\text{-}4$ ⇌ $C_{16}H_{32}OOH7\text{-}4{\dot{O}}_{2}$. Figure 9 compares the Gibbs free energy changes for these species before and after updating the GAVs. It shows that updating the GAVs results in a decrease of 2.294 kcal·mol^−1^ in the Gibbs free energy of ${\dot{C}}_{16}H_{33}\text{-}7$ and a decrease of 1.066 kcal·mol^−1^ in $C_{16}H_{33}\text{-}7{\dot{O}}_{2}$, while the Gibbs free energy of ${\dot{C}}_{16}H_{32}OOH7\text{-}4$ decreases by 4.578 kcal·mol^−1^. Conversely, the Gibbs free energy of $C_{16}H_{32}OOH7\text{-}4{\dot{O}}_{2}$ increases by 1.213 kcal·mol^−1^. This shift causes the equilibrium for ${\dot{C}}_{16}H_{33}\text{-}7$ ⇌ $C_{16}H_{33}\text{-}7{\dot{O}}_{2}$ and ${\dot{C}}_{16}H_{32}OOH7\text{-}4$ ⇌ $C_{16}H_{32}OOH7\text{-}4{\dot{O}}_{2}$ to shift to the left, reducing chain branching reactions. Simultaneously, the equilibrium $C_{16}H_{33}\text{-}7{\dot{O}}_{2}$ ⇌ ${\dot{C}}_{16}H_{32}OOH7\text{-}4$ shifts to the right. With the chain branching reaction of ${\dot{C}}_{16}H_{32}OOH7\text{-}4$ inhibited, the chain-propagation in its consumption pathway increases. Consequently, fuel reactivity decreases, and the simulated ignition delay time rises.

### 3.3. Chemical Kinetic Model Development

#### 3.3.1. Rate Rule Verification

**Figure 10:**
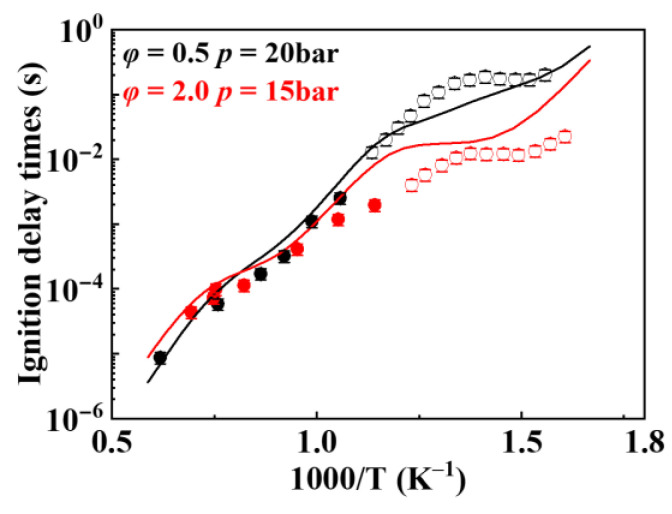
Comparison of simulated values with experimental data after initial mechanism adjustment (solid lines: simulated value; solid symbols: experimental data from shock tube [5]; open symbols: experimental data from rapid compression machine [4]).

The initial mechanism for *iso*-cetane constructed in this study selects NUIG-Mech1.3 [19,20] as the core mechanism, while the fuel-level sub-mechanism is developed using the $C_{6}$ rate rule proposed by Zhang et al. [14]. Figure 10 presents a comparison of the simulated ignition delay times from the initial mechanism with experimental data. It can be observed that the simulated ignition delay times under high-temperature conditions agree well with the experimental values. However, under low-temperature conditions, deviations exist between the simulated and experimental ignition delay times, indicating poor predictive performance under such conditions. The modifications were made in the selective rate constants based on a performance assessment of the final updated mechanism. The description of updated high and low-temperature reaction classes is included in the following sections.

#### 3.3.2. High-Temperature Chemistry

- Decomposition of fuel and fuel radicals

The reaction rates of unimolecular fuel decomposition and the decomposition of fuel radicals are crucial for high-temperature fuel oxidation. Unimolecular decomposition represents one of the primary fuel consumption pathways; enhancing its reaction rate can effectively reduce ignition delay times under high-temperature conditions. The decomposition rate of fuel radicals influences the oxidation rate of fuel in the high-temperature region; increasing this rate leads to a reduction in ignition delay times within these regions. The rate coefficients for unimolecular decomposition are assigned according to the identity of the fission products. In the present work, rate rules for channels yielding H atoms and fuel radicals were based on Allara et al. [28], those producing $\dot{C}H_{3}$ as the smallest fragment were based on Baulch et al. [29], and channels leading to other alkyl radicals were based on Mao et al. [30], with the corresponding pre-exponential factors (*A*) multiplied by a factor of 2 to enhance reactivity, improving the predictive performance of the mechanism under high-temperature conditions. The decomposition reaction of *iso*-cetane to $NEOC_{5}H_{11}$ and $AC_{11}H_{23}$ inhibits fuel oxidation, reduces the reaction rate constant, and increases the simulated ignition delay time. Therefore, the pre-exponential factor of this reaction was divided by 2 to enhance its reactivity, and this adjustment is based on the rate rule published in the literature [28].

**Figure 11:**
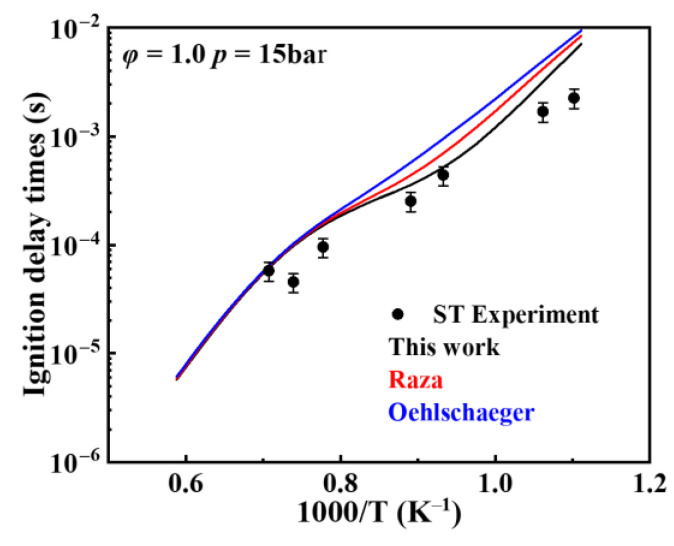
Comparison of simulated and experimental ignition delay times for the decomposition of fuel and its radicals at different rate constants (symbol: experimental data from shock tube [5]; black line: the present mechanism; red line: Raza mechanism [5]; blue line: Oehlschlaeger mechanism [3]).

For the decomposition reactions of fuel radicals, Oehlschlaeger et al. [3], Raza et al. [5], and the present work all referenced the work of Allara et al. [28]. Building upon this foundation, the present work generated Pressure-dependent Logarithmic interpolation (PLOG) rates and adjusted them based on different radical sites to enhance reaction rate constants, reduce ignition delay times, and improve the predictive performance of the mechanism. Figure 11 compares simulated and experimental values for the monomolecular cracking reaction of fuel and the cracking reaction of fuel radical compounds using reaction rate constants from different mechanisms. It is evident that the rates adjusted in the present work effectively enhance the predictive performance of the mechanism under high-temperature operating conditions. The recommended rate constant values for the high-temperature reaction classes are shown in Table 3. This table covers the Arrhenius parameters (pre-exponential factor *A*, temperature exponent *n*, and activation energy *Ea*) for high-temperature dominant reaction types such as fuel unimolecular decomposition and alkyl radical β-scission, forming the basis for constructing the high-temperature sub-mechanism of the model.

- Hydrogen abstraction reaction

**Figure 12:**
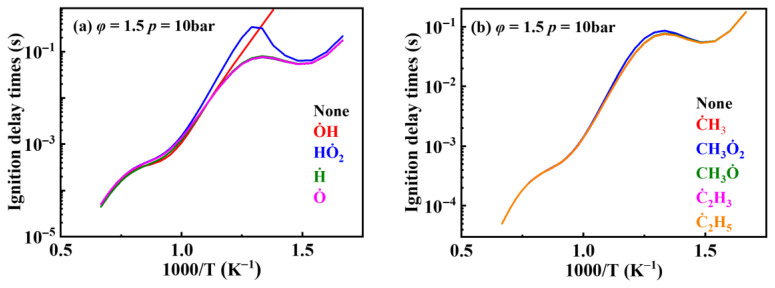
Influences of hydrogen abstraction reactions by different radicals on the simulated ignition delay times. (**a**) effects of hydrogen abstraction reactions by $\dot{O}H$, $H{\dot{O}}_{2}$, $\dot{H}$ and $\dot{O}$ individually shielded versus unshielded; (**b**) effects of hydrogen abstraction reactions by $\dot{C}H_{3}$, $CH_{3}{\dot{O}}_{2}$, $CH_{3}\dot{O}$, ${\dot{C}}_{2}H_{3}$ and ${\dot{C}}_{2}H_{5}$ individually shielded versus unshielded.

The hydrogen abstraction reaction, as the primary consumption pathway for *iso*-cetane, influences fuel reactivity under both high-temperature and low-temperature conditions [21]. Therefore, the selection of reaction rates significantly impacts the predictive capability of the mechanism. Different hydrogen abstraction reactions of radicals exhibit varying effects on simulated ignition delay times. In the present work, hydrogen abstraction reactions involving different radicals were individually shielded to simulate ignition delay times, thereby investigating their respective effects. The simulation results are shown in Figure 12, where “None” indicates that no reactions were shielded. It can be seen from Figure 12a that the hydrogen abstraction reactions by $\dot{O}H$ and $H{\dot{O}}_{2}$ have the greatest influence on the simulated ignition delay time of *iso*-cetane. In Figure 12b, after individually shielding the hydrogen abstraction reactions of five radicals, the simulated ignition delay times are essentially coincident, with only a slight difference observed when the hydrogen abstraction reaction of $CH_{3}{\dot{O}}_{2}$ is shielded. This indicates that the hydrogen abstraction reaction of $CH_{3}{\dot{O}}_{2}$ has a certain impact on the simulated value. The recommended rate values for hydrogen abstraction reactions of the fuel are shown in Table 4. In this table, the rate constants for hydrogen abstraction by different radicals (e.g., $H{\dot{O}}_{2}$, $CH_{3}{\dot{O}}_{2}$, etc.) are listed separately according to the type of carbon site (primary, secondary, or tertiary) from which the hydrogen atom is abstracted. These reactions are key steps in fuel consumption and radical transfer.

**Figure 13:**
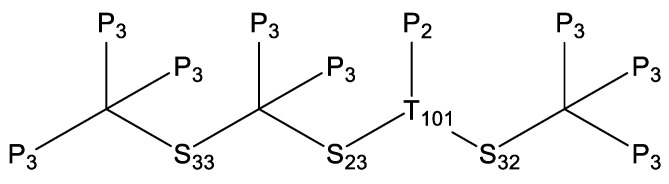
Structure and carbon position of *iso*-cetane based on the NNN method.

**Figure 14:**
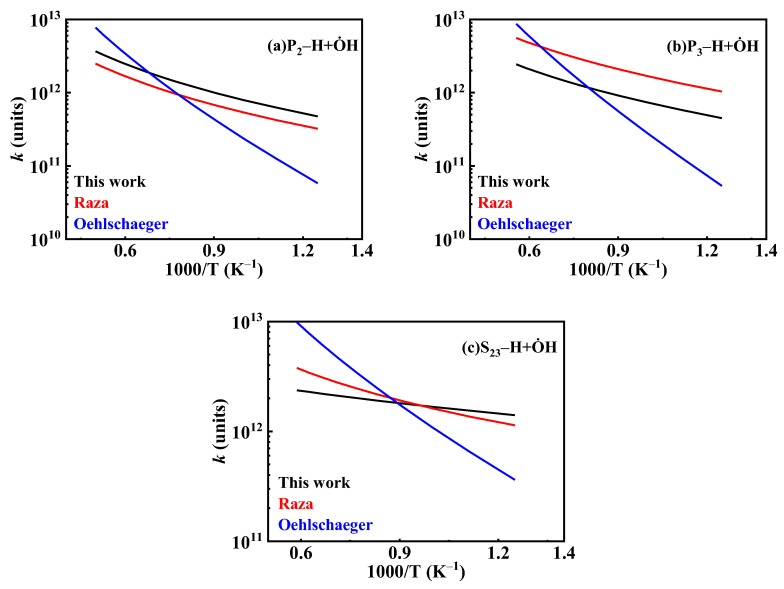
Comparisons of rate constants for hydrogen extraction reactions from *iso*-cetane by $\dot{O}H$. (**a**) hydrogen abstraction from the $P_{2}$ site; (**b**) hydrogen abstraction from the $P_{3}$ site; (**c**) hydrogen abstraction from the $S_{23}$ site.

The existing $\dot{O}H$ hydrogen abstraction reaction rate constants for *iso*-cetane primarily originate from studies by Cohen [31], Sivaramakrishnan et al. [32], and Badra et al. [33]. Cohen [31] estimated the reaction constants between $\dot{O}H$ and alkanes based on transition state theory (TST) combined with experimental data. Sivaramakrishnan et al. [32] developed a more accurate group contribution model based on experimental data to predict $\dot{O}H$ hydrogen abstraction reaction rate constants. Badra et al. [33] proposed a model applicable to predicting $\dot{O}H$ hydrogen abstraction reaction rate constants for any branched alkane, integrating experimental and literature data. In the present work, the $\dot{O}H$ hydrogen abstraction reaction was based on the next nearest neighbor (NNN) method. Figure 13 shows the positions of its carbon atoms based on the NNN method. The NNN method considers the environment of the central carbon atom and its next-nearest-neighbor carbon atoms, further subdividing the different types of carbon sites (primary, secondary, and tertiary) in *iso*-cetane into sub-categories with distinct local chemical environments, thereby enabling a more accurate estimation of the rate constants for hydrogen abstraction reactions at different sites. Reaction rate constants for some sites ($P_{2}$, $P_{3}$, $S_{23}$) were updated according to the study of Sivaramakrishnan et al. [32], while those for other sites ($S_{23}$, $S_{33}$, $T_{101}$) referenced the study of Badra et al. [33]. The comparison of $\dot{O}H$ hydrogen abstraction reaction rate constants is shown in Figure 14.

**Figure 15:**
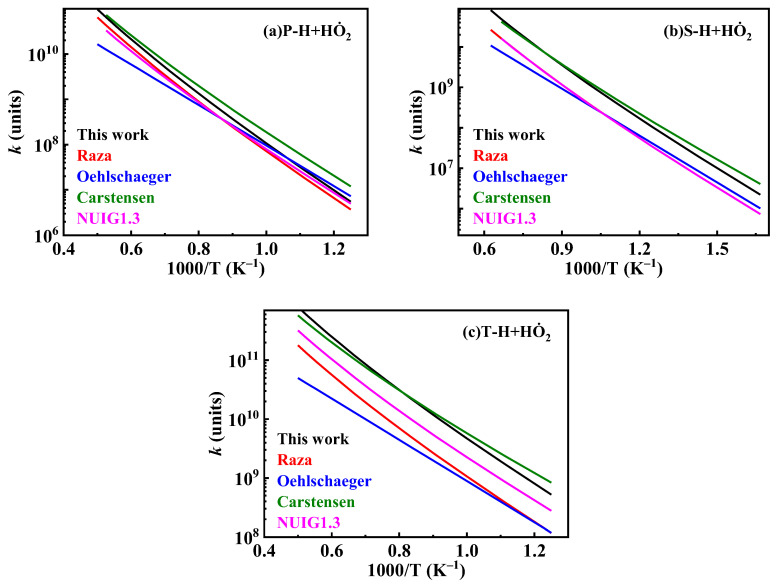
Comparisons of rate constants for hydrogen extraction reactions from *iso*-cetane by $H{\dot{O}}_{2}$. (**a**) hydrogen abstraction from primary sites; (**b**) hydrogen abstraction from secondary sites; (**c**) hydrogen abstraction from tertiary sites.

The existing $H{\dot{O}}_{2}$ hydrogen abstraction reaction rate constants for *iso*-cetane primarily stem from studies by Walker [34] and Aguilera-Iparraguirre et al. [35]. Walker [34] experimentally determined the reaction rate constants for $H{\dot{O}}_{2}$ hydrogen extraction from alkanes. Aguilera-Iparraguirre et al. [35] employed quantum chemical methods to investigate the reaction energy barriers and rate constants for $H{\dot{O}}_{2}$ hydrogen abstraction from methane and $C_{2}–C_{4}$ alkanes. In the present mechanism, the rate constants for $H{\dot{O}}_{2}$ hydrogen abstraction are based on the results of Aguilera-Iparraguirre et al. [35], while simultaneously multiplying the pre-exponential factor by 1.5 to enhance reactivity. The pre-exponential factor was multiplied by 1.5 based on the combined uncertainty reported in the study by Aguilera-Iparraguirre et al. [35]. The comparison of $H{\dot{O}}_{2}$ hydrogen abstraction reaction rate constants is shown in Figure 15.

**Figure 16:**
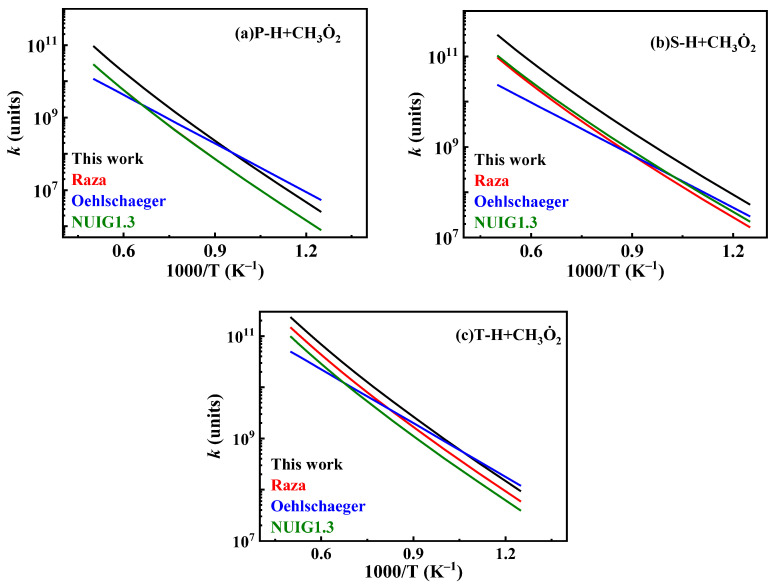
Comparisons of rate constants for hydrogen extraction reactions from *iso*-cetane by $CH_{3}{\dot{O}}_{2}$. (**a**) hydrogen abstraction from primary sites; (**b**) hydrogen abstraction from secondary sites; (**c**) hydrogen abstraction from tertiary sites.

The current hydrogen abstraction reaction rate constants for $CH_{3}{\dot{O}}_{2}$ for *iso*-cetane primarily stem from studies by Curran et al. [10] and Healy et al. [36]. While developing the mechanism for *iso*-octane, Curran et al. [10] analogized the hydrogen abstraction reactions of $H{\dot{O}}_{2}$ and $CH_{3}{\dot{O}}_{2}$, setting the $CH_{3}{\dot{O}}_{2}$ reaction rate constants at 0.72 times that of $H{\dot{O}}_{2}$ extraction. Healy et al. [36], while investigating the isobutane mechanism, estimated the $CH_{3}{\dot{O}}_{2}$ hydrogen abstraction rate by analogy with the $H{\dot{O}}_{2}$ hydrogen abstraction reactions. In the present mechanism, the rate constants for $CH_{3}{\dot{O}}_{2}$ hydrogen abstraction are based on the work of Healy et al. [36], while simultaneously multiplying the pre-exponential factor for hydrogen abstraction on P and S alkyl groups by 2 to enhance reaction activity. Reaction rate constants for hydrogen abstraction reactions between other radicals ($\dot{H}$, $\dot{O}$, $CH_{3}\dot{O}$, ${\dot{C}}_{2}H_{3}$, ${\dot{C}}_{2}H_{5}$) and fuels adopt those from the Oehlschlaeger mechanism [3]. The reaction rate constants for hydrogen extraction between $\dot{C}H_{3}$ and fuels adopt that from the Raza mechanism [5]. The hydrogen abstraction reaction rate constants of $CH_{3}{\dot{O}}_{2}$ are compared as shown in Figure 16.

**Table 4:** Recommended values for the reaction rate constants of *iso*-cetane hydrogen abstraction. P = Primary carbon, S = Secondary carbon, T = Tertiary carbon. Rate constant in Arrhenius form (*k* = *AT^n^* exp(*Ea*/*RT*)); units are cm^3^, K, mol, s, and kJ.

Radical	Site	*A*	*n*	*Ea*	Source
$H{\dot{O}}_{2}$	P	10.2	3.59	1.716 × 10^4^	Aguilera-Iparraguirre et al. [35]
	S	94.8	3.37	1.372 × 10^4^	
	T	1922	3.01	1.209 × 10^4^	
$CH_{3}{\dot{O}}_{2}$	P	0.462	3.97	1.828 × 10^4^	Healy et al. [36]
	S	10.18	3.58	1.418 × 10^4^	
	T	206	3.12	1.319 × 10^4^	

#### 3.3.3. Low-Temperature Chemistry

The autoignition of fuels is closely related to chain-branching reaction mechanisms [37]. Under low-temperature conditions (<900 K), low-temperature oxidation isomerization pathways ($\dot{R}$ + $O_{2}$ = $R{\dot{O}}_{2}$, $R{\dot{O}}_{2}$ = $\dot{Q}OOH$, $\dot{Q}OOH$ + $O_{2}$ = ${\dot{O}}_{2}QOOH$, ${\dot{O}}_{2}QOOH$ = KHP + $\dot{O}H$, ${\dot{O}}_{2}QOOH$ = $\dot{P}{\left(OOH\right)}_{2}$, and $\dot{P}{\left(OOH\right)}_{2}$ decomposition) become dominant chain-branching pathways. However, as temperature increases, the three chain-propagation pathways of $\dot{Q}OOH$ ($\dot{Q}OOH$ = cyclic ether + $\dot{O}H$, $\dot{Q}OOH$ = olefin + $H{\dot{O}}_{2}$, $\dot{Q}OOH$ = olefin + C=O + $\dot{O}H$) gradually increase, suppressing the low-temperature chain-branching pathways. This leads to a decrease in reaction activity with rising temperature, a phenomenon known as the NTC effect [38]. As noted in the introduction, existing *iso*-cetane mechanisms exhibit prediction deviations in the NTC region. Therefore, it is necessary to discuss specific reaction rate constants within the low-temperature mechanism.

- $\dot{R}$ + $O_{2}$ and $\dot{Q}OOH$ + $O_{2}$

Under low-temperature conditions, the oxidation reactions of fuel radical $\dot{R}$ and $\dot{Q}OOH$ are crucial, significantly influencing the low-temperature oxidation rate of fuels. These reactions are indispensable in the low-temperature chain-branching pathway. Increasing their reaction rate constants can enhance the contribution of this pathway and reduce the ignition delay times of *iso*-cetane under low-temperature conditions [38]. Miyoshi [39] calculated the reaction rate constant of $\dot{R}$ + $O_{2}$ using the variational transition state theory (VTST) and the Reiss–Ramberg–Cassel–Marcus theory/primary equation. Goldsmith et al. [40] calculated the reaction rate constants for the P and S alkyl groups in the reaction $\dot{R}$ + $O_{2}$ using variable reaction coordinate transition state theory (VRC-TST). Villano et al. [41] derived the reverse reaction rate constants for $\dot{R}$ + $O_{2}$ from the equilibrium constant of the reaction and relevant literature data. In the present mechanism, the rate constants for $\dot{R}$ + $O_{2}$ are based on the values calculated by Miyoshi [39], with the pre-exponential factors scaled by 0.8. Goldsmith et al. [40] found that the reaction rate constant for $\dot{Q}OOH$ + $O_{2}$ should be half that of $\dot{R}$ + $O_{2}$. Therefore, the reaction rate constant for $\dot{Q}OOH$ + $O_{2}$ in the present work is adjusted to half that of the $\dot{R}$ + $O_{2}$ reaction. In the kinetic mechanism proposed by Oehlschlaeger et al. [3], the reaction rate constant for $\dot{Q}OOH$ + $O_{2}$ is half that of the aforementioned $\dot{R}$ + $O_{2}$ reaction. However, Raza et al. [5] multiplied the pre-exponential factor by 3 based on the study of Oehlschlaeger et al. [3] to match the experimental data. Table 5 and Table 6 present the recommended reaction rate values for the oxidation reactions of alkyl radicals and hydroperoxyalkyl radicals, respectively, during the oxidation of *iso*-cetane. According to the type of carbon atom (primary, secondary, or tertiary) from which the hydrogen atom is abstracted, the pre-exponential factor (*A*), temperature exponent (*n*), and activation energy (*Ea*) are listed. These values can be directly used to construct or update the relevant reaction rate constants in the low-temperature oxidation sub-mechanism of *iso*-cetane.

**Figure 17:**
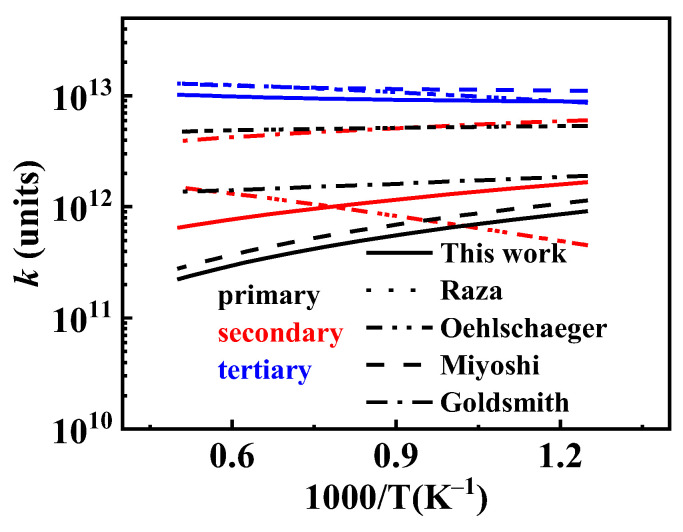
Comparison of rate constants for $\dot{R}$ + $O_{2}$ between the present work and the literature.

**Figure 18:**
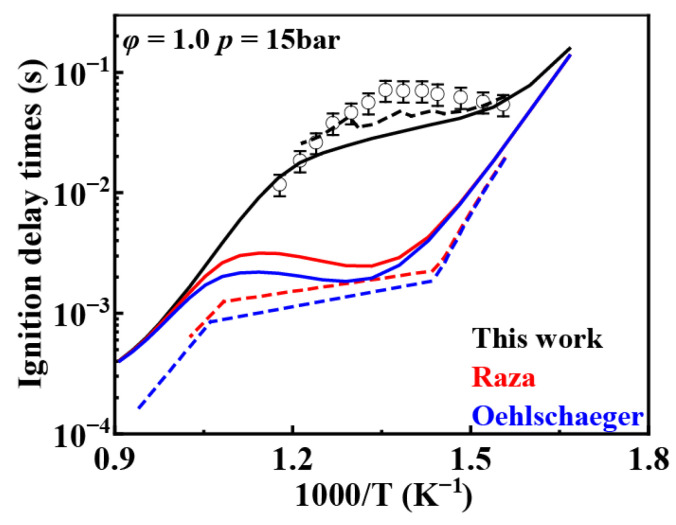
Comparison of simulated and experimental ignition delay times for the oxidation reaction of fuel radical $\dot{R}$ and $\dot{Q}OOH$ at different rate constants (open symbol: experimental data from rapid compression machine [4]; solid lines: constant volume simulation; dash lines: variable volume simulation).

Figure 17 presents the comparison results for the rate constants for $\dot{R}$ + $O_{2}$ between the present work and the literature. The reaction rate constants for $\dot{R}$ + $O_{2}$ on P and S alkyl groups in the mechanisms developed by Oehlschlaeger et al. [3] and Raza et al. [5] differ from those in the mechanism of the present work. The updated rate constants in the present work are reduced, lowering the oxidation rate of fuel under low-temperature conditions, increasing the ignition delay times, and improving the predictive accuracy of the mechanism. Figure 18 compares simulated and experimental values for reactions $\dot{R}$ + $O_{2}$ and $\dot{Q}OOH$ + $O_{2}$ using different mechanisms. The results indicate that the simulated values from the selected rate mechanism in the present work best match the experimental data. Furthermore, as temperature increases, the discrepancies between simulated values from the three rate mechanisms gradually diminish. Above 1000 K, the simulated values from all three mechanisms become essentially consistent. This demonstrates that the oxidation reactions of fuel radical $\dot{R}$ and $\dot{Q}OOH$ progressively reduce their influence on ignition delay times with increasing temperature, becoming negligible beyond 1000 K.

- $R{\dot{O}}_{2}$ ⇌ $\dot{Q}OOH$ isomerization

Research indicates that the isomerization of $R{\dot{O}}_{2}$ ($R{\dot{O}}_{2}$ ⇌ $\dot{Q}OOH$) competes with the concerted elimination reaction ($R{\dot{O}}_{2}$ = alkene + $H{\dot{O}}_{2}$), promoting the formation of the NTC region in alkanes. The $\dot{Q}OOH$ generated from $R{\dot{O}}_{2}$ isomerization significantly influences the chain-branching pathway. Its rate constant is primarily affected by the type of carbon atom bonded to the migrating H atom, and is also related to the alkyl structure [42]. Curran et al. [10] summarized new $R{\dot{O}}_{2}$ isomerization reaction rate constants by referencing works by Benson [43], Pollard [44], and Bozzelli and Pitz [45]. Villano et al. [46] calculated the reaction rate constants for $R{\dot{O}}_{2}$ ⇌ $\dot{Q}OOH$ using the complete basis set-quadratic becke3 (CBS-QB3) method. Sharma et al. [42] calculated relevant reaction rate constant for $R{\dot{O}}_{2}$ ⇌ $\dot{Q}OOH$ based on CBS-QB3 theory, finding that the reaction rate constant or $R{\dot{O}}_{2}$ ⇌ $\dot{Q}OOH$ is primarily influenced by the type of carbon atom bonded to the migrating $\dot{H}$ atom. In the present mechanism, the rate constant for $R{\dot{O}}_{2}$ ⇌ $\dot{Q}OOH$ is based on the work of Sharma et al. [42], with the pre-exponential factor scaled by 2.5 to enhance reactivity. The recommended rate values for isomerization reactions are shown in Table 7, including the recommended reaction rate values for the isomerization of alkylperoxy radicals ($R{\dot{O}}_{2}$) to hydroperoxyalkyl radicals ($\dot{Q}OOH$). In this table, the pre-exponential factor (*A*), temperature exponent (*n*), and activation energy (*Ea*) are listed separately according to the ring size of the transition state (5-, 6-, 7-, or 8-membered) and the type of hydrogen atom being transferred (primary, secondary, or tertiary). These values can be directly used to construct or update the low-temperature oxidation sub-mechanism of *iso*-cetane.

**Figure 19:**
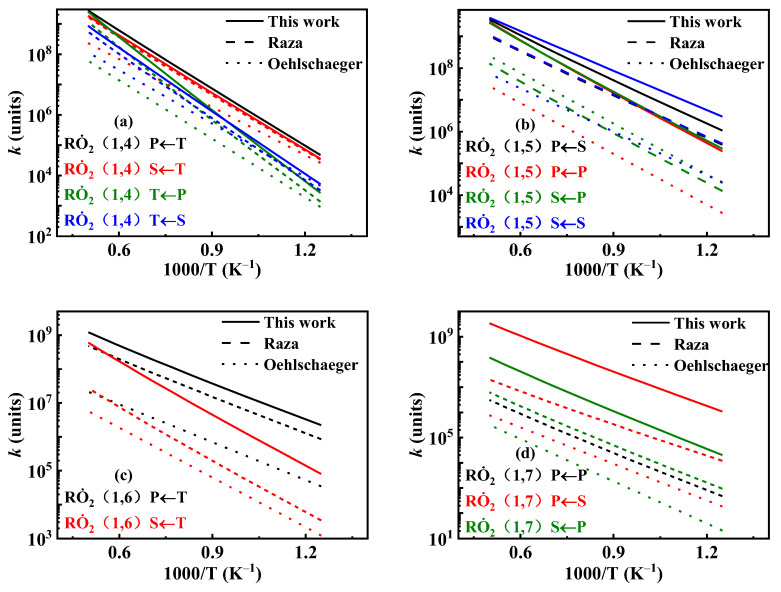
Comparisons of rate constants for $R{\dot{O}}_{2}$ ⇌ $\dot{Q}OOH$. (**a**) 1,4-H shift; (**b**) 1,5-H shift; (**c**) 1,6-H shift; (**d**) 1,7-H shift.

**Figure 20:**
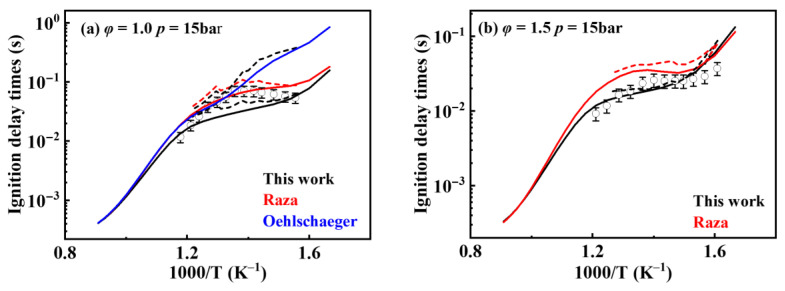
Comparisons of simulated and experimental ignition delay times for $R{\dot{O}}_{2}$ isomerization reactions at different rate constants (open symbols: experimental data from rapid compression machine [4]; solid lines: constant volume simulation; dash lines: variable volume simulation). (**a**) *φ* = 1.0 and *p* = 15 bar; (**b**) *φ* = 1.5 and *p* = 15 bar.

Figure 19 shows the comparison of reaction rate constants for the $R{\dot{O}}_{2}$ isomerization reaction under different mechanisms. It can be observed that the rate constant in the mechanism of Oehlschlaeger et al. [3] exhibits significant discrepancies compared to other studies. Figure 20 shows the comparison between simulated and experimental values for $R{\dot{O}}_{2}$ isomerization using different mechanisms. As seen in Figure 20a, the simulated values using the reaction rate constants of Oehlschlaeger et al. [3] approach a straight line and lack an NTC region, while simulations employing the rate constants of Raza and the present work show better agreement with experimental data. Specifically, under conditions *φ* = 1.0 and *p* = 15 bar, the reaction rate constant of Raza et al. [5] yields higher correlation with experimental values. However, Figure 20b reveals that as the equivalence ratio increases, the correlation between the rates from the Raza et al. [5] mechanism and experimental data significantly deteriorates, whereas mechanism of the present work maintains superior agreement.

- Concerted elimination reaction

Although the concerted elimination reaction contributes a relatively small proportion of the reaction pathway, it significantly influences the ignition delay times under low-temperature conditions. This reaction produces relatively stable olefin and low-reactivity $H{\dot{O}}_{2}$, thereby suppressing oxidative reactivity and increasing ignition delay times. The rate constant for the concerted elimination reaction primarily depends on the position of the removed hydrogen atom and the type of carbon atom bonded to the -COO group [41]. Villano et al. [41] employed the CBS-QB3 method and transition state theory to calculate the reaction rate constants for $R{\dot{O}}_{2}$ = olefin + $H{\dot{O}}_{2}$, summarizing corresponding rate rules for different mechanisms. Snisiriwat et al. [47] utilized the CBS-QB3 method to compute reaction rate constants for $R{\dot{O}}_{2}$-related reaction classes in the *iso*-octane mechanism, including the concerted elimination reaction. The reaction rate constants of the concerted elimination reaction in the present work are based on the study of Villano et al. [41], multiplying the pre-exponential factor of the P, S carbon reaction by 2. The recommended rate values for concerted elimination reactions are shown in Table 8, including the recommended rate values for the concerted elimination reactions of $R{\dot{O}}_{2}$ and ${\dot{O}}_{2}QOOH$ to produce alkenes and $H{\dot{O}}_{2}$. These parameters are essential for accurately modeling the competition between chain-branching and chain-propagation pathways in the negative temperature coefficient (NTC) region.

**Figure 21:**
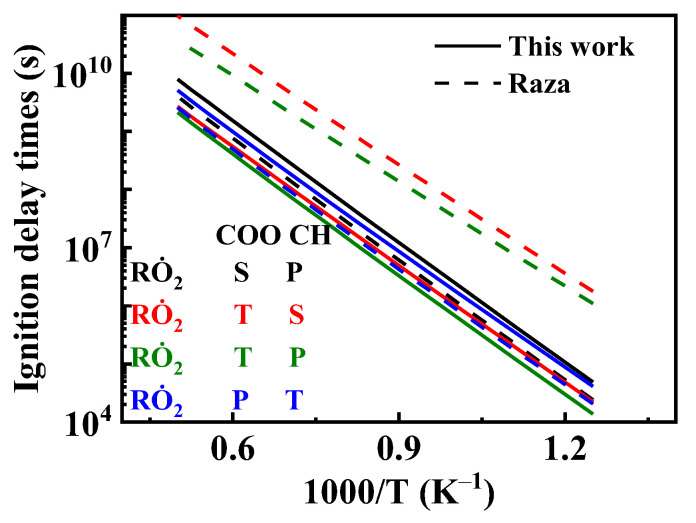
Comparison of reaction rate constants for concerted elimination reaction.

**Figure 22:**
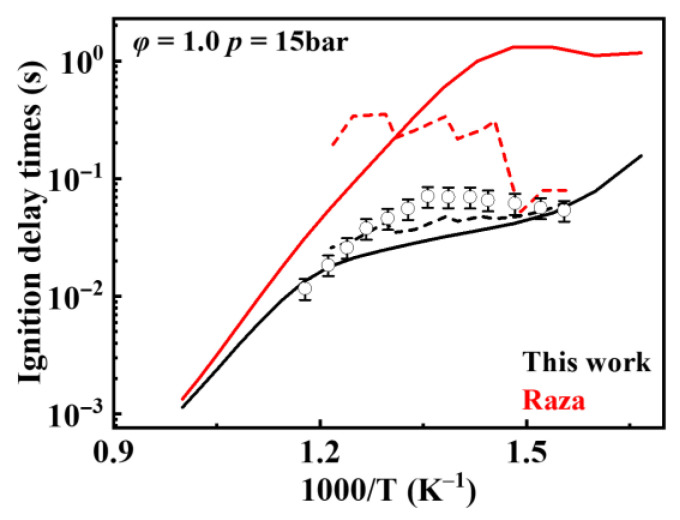
Comparison of simulated and experimental ignition delay times for concerted elimination reactions at different rate constants (open symbol: experimental data from rapid compression machine [4]; solid lines: constant volume simulation; dash lines: variable volume simulation).

Figure 21 shows a comparison of rate constants for concerted elimination reactions. The reaction rate constants from Raza et al. [5] differ from site to site because each site was taken from a different study. Figure 22 presents a comparison between simulated values obtained using reaction rate constants from different mechanisms and experimental values for the concerted elimination reaction. It can be observed that simulation results based on the reaction rate constants from Raza et al. [5] show poor agreement with experimental data under low-temperature conditions, whereas simulation results using the rate derived in the present work exhibit relatively better agreement with experimental data.

- $\dot{Q}OOH$ ⇌ cyclic ether + $\dot{O}H$

The cyclic ether formation reaction of $\dot{Q}OOH$ ($\dot{Q}OOH$ ⇌ cyclic ether + $\dot{O}H$) is significant for the low-temperature oxidation process of alkane fuels. This reaction belongs to the chain-propagation pathway, competing with the chain-branching pathway. Inhibiting the low-temperature chain-branching pathway and increasing its reaction rate constants can decrease the proportion of the chain-branching pathway, thereby increasing the ignition delay times in the NTC region. This has a significant impact on the ignition delay times in the NTC region of *iso*-cetane. Villano et al. [46] calculated the reaction rate constants for representative $\dot{Q}OOH$ reactions using the CBS-QB3 method. Their study revealed that the activation energy for cyclic ether formation is primarily influenced by the reaction enthalpy. Miyoshi [39] systematically calculated the reaction rate constants for cyclic ether formation reaction using the CBS-QB3 method, revealing that the activation energy depends not only on the type of carbon atom bonded to the -OOH group but also on the types of carbon atoms within the ring structure. Wijaya et al. [48] employed CBS and scaled frequency treatment (SFT) methods to calculate the reaction rate constants for the cyclic ether formation reaction and summarized the estimation rules for these rates. The reaction rate constants for the cyclic ether formation reaction in the mechanism constructed in the present work are based on the *iso*-cetane mechanism developed by Heng [49]. Adjustments were made for specific reactions, with details and adjustment factors listed in Table 9.

**Figure 23:**
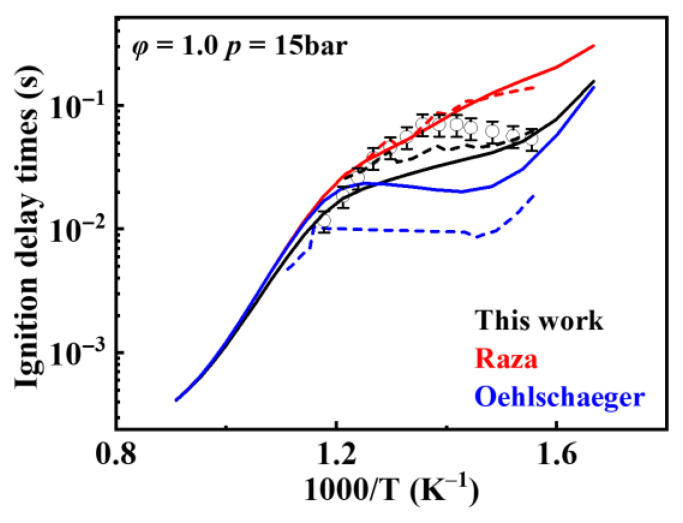
Comparison of simulated and experimental ignition delay times for cyclic ether formation reactions with different rate constants (open symbol: experimental data from rapid compression machine [4]; solid lines: constant volume simulation; dash lines: variable volume simulation).

Figure 23 shows the comparison between simulated and experimental values obtained using different reaction rate constant mechanisms for the cyclic ether formation reaction. It can be observed that the simulated values using the rate of Oehlschlaeger et al. [3] show poor agreement with experimental data. In contrast, the simulated values using the rate of Raza et al. [5] exhibit better agreement than those using Oehlschlaeger et al. [3], though they do not exhibit an NTC region. The simulated values using the rate proposed in the present work show excellent agreement with experimental data. Furthermore, the simulated values from all three rates become essentially consistent above approximately 830 K.

- Ketohydroperoxide formation and decomposition

The reactions for formation of ketohydroperoxide (${\dot{O}}_{2}QOOH$ = KHP + $\dot{O}H$) and its decomposition are crucial for low-temperature oxidation of alkanes. Ketohydroperoxide generates two radicals through decomposition, representing a typical chain-branching reaction that enhances fuel oxidation activity, increases oxidation rates, and reduces ignition delay times in low-temperature regions. Curran et al. [10] proposed that the pre-exponential factor for $\dot{H}$ atom migration in the ${\dot{O}}_{2}QOOH$ radical is one-third that of the corresponding reaction rate constant for the $R{\dot{O}}_{2}$ radical, with an activation energy 3 kcal mol^−1^ lower than that of the $R{\dot{O}}_{2}$ radical. Sharma et al. [42] calculated reaction rate constants for representative species in the ${\dot{O}}_{2}QOOH$ radical isomerization reaction using CBS-QB3 and B3LYP/CBSB7. The reaction rate constants for the ketohydroperoxide formation step in the mechanism developed in the present work also referenced the calculated values of Sharma et al. [42], while doubling the pre-exponential factor to enhance reactivity. For the ketohydroperoxide decomposition reaction in the present work, the activation energy for the ketohydroperoxide decomposition step in the mechanism of Oehlschlaeger et al. [3] was reduced by 1500 cal, as referenced from the mechanism of Heng [49], to enhance reactivity. The recommended rate constant values for the formation reactions of ketohydroperoxide are shown in Table 10.

**Figure 24:**
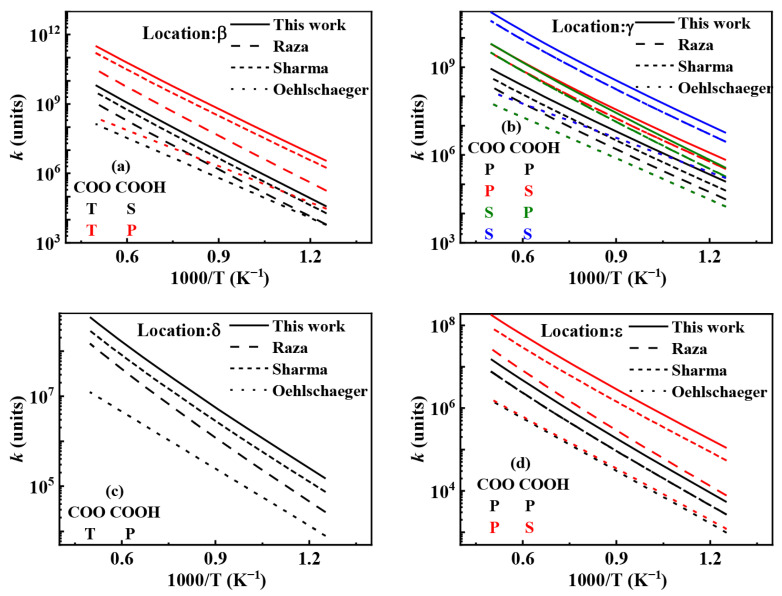
Comparisons of rate constants for the ketohydroperoxide formation reactions. (**a**) β-location; (**b**) γ-location; (**c**) δ-location; (**d**) ε-location.

**Figure 25:**
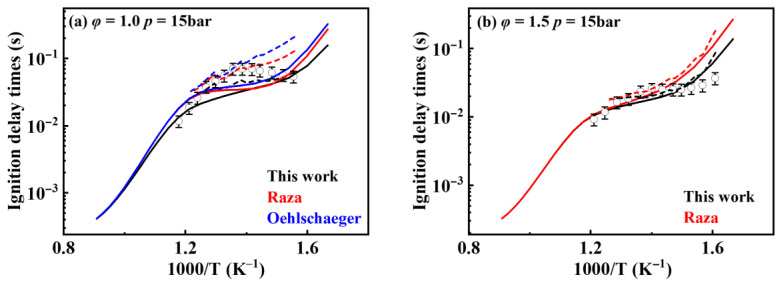
Comparisons of simulated and experimental ignition delay times for ketohydroperoxide formation and decomposition reactions at different rate constants (open symbols: experimental data from rapid compression machine [4]; solid lines: constant volume simulation; dash lines: variable volume simulation). (**a**) *φ* = 1.0, *p* = 15 bar; (**b**) *φ* = 1.5, *p* = 15 bar.

Figure 24 shows the comparison of reaction rate constants for ketohydroperoxide formation under different mechanisms. The reaction rate constants in the present work are higher than those reported in other studies, and the rate under the mechanism of Oehlschlaeger et al. [3] is consistently lower than the rates reported in other studies. Figure 25 presents the comparison between simulated and experimental values for hydrogen peroxone formation and cleavage reactions using different mechanisms. Figure 25a indicates that simulations using the reaction rate constants of Oehlschlaeger et al. [3] show poor agreement with experimental data and fail to reproduce the NTC region. The simulated values using the Raza reaction rate constants show better agreement with experimental data and exhibit higher consistency relative to the rate proposed in the present work. However, as shown in Figure 25b, the agreement between simulated and experimental values using the reaction rate constants of Raza et al. [5] deteriorates significantly with increasing equivalence ratio, whereas the simulated values based on the mechanism proposed in the present work maintain good agreement with experimental data.

- Formation and decomposition of $\dot{P}{\left(OOH\right)}_{2}$

**Figure 26:**
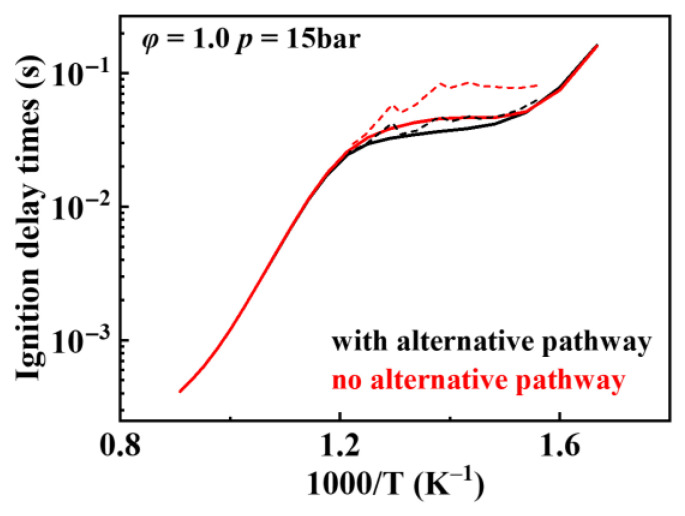
Comparison of simulated ignition delay times before and after adding the relevant path (solid lines: constant volume simulation; dash lines: variable volume simulation).

In previously developed mechanisms [3,4,5], the sole reaction pathway for peroxyhydroperoxyalkyl (${\dot{O}}_{2}QOOH$) involves intramolecular hydrogen atom migration to form hydrogen peroxone and $\dot{O}H$. However, research [12] reveals that peroxyhydroperoxyalkyl (${\dot{O}}_{2}QOOH$) competes with this pathway through alternative routes: isomerization yielding $\dot{P}{\left(OOH\right)}_{2}$ and concerted elimination reactions. The concerted elimination reaction produces olefin and $H{\dot{O}}_{2}$, which inhibit oxidative reaction activity. The formation of $\dot{P}{\left(OOH\right)}_{2}$ competes with the KHP formation pathway, and this competition significantly influences the ignition delay times in the low-temperature region. The consumption pathway of $\dot{P}{\left(OOH\right)}_{2}$ also affects the ignition delay times in the low-temperature region. Wang et al. [50,51] demonstrated that the consumption pathway of $\dot{P}{\left(OOH\right)}_{2}$ belongs to a chain-branching pathway, reducing the simulated ignition delay times under low-temperature conditions. Therefore, the present work incorporates additional reactions into the existing detailed mechanism: (1) isomerization of ${\dot{O}}_{2}QOOH$ to $\dot{P}{\left(OOH\right)}_{2}$, (2) concerted elimination of ${\dot{O}}_{2}QOOH$ to form EROOH and $H{\dot{O}}_{2}$, and (3) consumption of $\dot{P}{\left(OOH\right)}_{2}$. The rate of hydroperoxyalkyl radical isomerization was based on the reaction rate constants of $R{\dot{O}}_{2}$ ⇌ $\dot{Q}OOH$; the rate constant for the concerted elimination of hydroperoxyalkyl radical was based on that of $R{\dot{O}}_{2}$; and the reaction rate constant for $\dot{P}{\left(OOH\right)}_{2}$ consumption was based on the corresponding $\dot{Q}OOH$ consumption rate. Figure 26 illustrates the effect of adding relevant reaction pathways on simulated ignition delay times. It is evident that the added reaction pathways generally promote the oxidation of fuel under low-temperature conditions, thereby reducing simulated ignition delay times. The recommended rate values for the formation and consumption reactions of $\dot{P}{\left(OOH\right)}_{2}$ are shown in Table 11 and Table 12. The formation and consumption of alkyl-dihydroperoxides release additional $\dot{O}H$ radicals and promote chain branching under low-temperature conditions. These reactions are crucial for accurately capturing the multi-stage ignition behavior and the negative temperature coefficient (NTC) region of *iso*-cetane.

In the present work, a detailed kinetic mechanism for *iso*-cetane is developed by updating the thermodynamic data and the core sub-mechanism, supplementing previously missing reaction classes, and revising the reaction rate constants based on recent literature. The resulting mechanism comprises 4541 species and 18,359 elementary reaction steps. The mechanism, along with the associated thermodynamic, is available in the Files S1 and S2 (Supplementary Materials).

## 4. Conclusions

In the present work, the reaction categories and thermodynamic data of *iso*-cetane were investigated. A comparative analysis of the reaction rate constants under existing mechanisms was conducted. Based on the aforementioned research, a chemical kinetic model for *iso*-cetane was constructed and validated. Reaction pathway and sensitivity analyses were performed on the ignition process. The main conclusions are as follows:

(1) Updated thermodynamic data for *iso*-cetane within the kinetic model based on the latest group values. The model with updated thermodynamic data shows improved prediction accuracy for the ignition delay time of *iso*-cetane in the NTC region.
(2) Investigated the reaction types and reaction rate constants of *iso*-cetane and its intermediates. This includes fuel cracking reactions, hydrogen extraction reactions, fuel radical cracking reactions, fuel radical oxidation, isomerization, synergistic elimination reactions, and reactions leading to the formation of cyclic ethers and ketohydroperoxide. Additionally, supplementary reactions were included, such as those related to the formation and consumption of $\dot{P}{\left(OOH\right)}_{2}$.
(3) Developed a chemical kinetic model for *iso*-cetane comprising 4541 species and 18,359 elementary reaction steps. The ignition delay times of the present model were validated under conditions of equivalence ratios ranging from 0.5 to 2.0, pressures from 10 to 20 bar, and temperatures from 600 to 1600 K. Additionally, the concentration changes of key species during the oxidation process were verified under equivalence ratios from 0.5 to 2.0, a pressure of 10 atm, and temperatures from 770 to 1070 K. Results indicate improved predictive performance compared to previous models.
(4) Reaction pathway analysis and sensitivity analysis of the ignition process were conducted at an equivalence ratio of 1.0, pressure of 15 bar, and temperatures of 600 K and 700 K. Findings reveal that the formation of the *iso*-cetane NTC region correlates with chain branching in the low-temperature region and competition among chain-propagation pathways.

## Figures and Tables

**Table 1:** Reaction categories included in the *iso*-cetane mechanism developed in the present work.

Number	Reaction Class	Number	Reaction Class
R1	Decomposition of fuel	R18	$R{\dot{O}}_{2}$ + $H_{2}O_{2}$ = ROOH + $H{\dot{O}}_{2}$
R2	H atom abstraction from *iso*-cetane	R19	$R{\dot{O}}_{2}$ + $CH_{3}{\dot{O}}_{2}$ = $R\dot{O}$ + $CH_{3}\dot{O}$ + $O_{2}$
R3	Alkyl radical decomposition	R20	$R{\dot{O}}_{2}$ + $R{\dot{O}}_{2}$ = $R\dot{O}$ + $R\dot{O}$ + $O_{2}$
R4	$\dot{R}$ + $O_{2}$ = olefins + $H{\dot{O}}_{2}$	R21	ROOH = $R\dot{O}$ + $\dot{O}H$
R5	Alkyl radical isomerization	R22	$R{\dot{O}}_{2}$ + RH = ROOH + $\dot{R}$
R6	H atom abstraction from olefins	R23	Alkoxy radical decomposition
R7	Addition reactions with olefins	R24	$\dot{Q}OOH$ = cyclic ether + $\dot{O}H$
R8	Alkenyl radical decomposition	R25	$\dot{Q}OOH$ = olefins + $H{\dot{O}}_{2}$
R9	Olefins decomposition	R26	Beta decomposition of $\dot{Q}OOH$
R10	Retro-ene decomposition	R27	$\dot{Q}OOH$ + $O_{2}$ = ${\dot{O}}_{2}QOOH$
R11	$\dot{R}$ + $O_{2}$ = $R{\dot{O}}_{2}$	R28	${\dot{O}}_{2}QOOH$ = KHP + $\dot{O}H$
R12	$\dot{R}$ + $R{\dot{O}}_{2}$ = $R\dot{O}$ + $R\dot{O}$	R29	Decomposition of KHP
R13	$\dot{R}$ + $H{\dot{O}}_{2}$ = $R\dot{O}$ + $\dot{O}H$	R30	Reactions of cyclic ethers
R14	$\dot{R}$ + $CH_{3}{\dot{O}}_{2}$ = $R\dot{O}$ + $CH_{3}\dot{O}$ + $O_{2}$	R31	${\dot{O}}_{2}QOOH$ = EROOH + $H{\dot{O}}_{2}$
R15	$R{\dot{O}}_{2}$ = $\dot{Q}OOH$	R32	${\dot{O}}_{2}QOOH$ = $\dot{P}{\left(OOH\right)}_{2}$
R16	$R{\dot{O}}_{2}$ = olefins + $H{\dot{O}}_{2}$	R33	Decomposition of $\dot{P}{\left(OOH\right)}_{2}$
R17	$R{\dot{O}}_{2}$ + $H{\dot{O}}_{2}$ = ROOH + $O_{2}$	R34	Decomposition of EROOH

**Table 2:** Structures of the major species in the low-temperature oxidation of *iso*-cetane.

Species	Structure	Species	Structure
${\dot{C}}_{16}H_{33}\text{-}2$	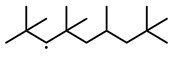	${\dot{C}}_{16}H_{32}OOH5\text{-}3$	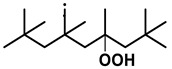
${\dot{C}}_{16}H_{33}\text{-}5$	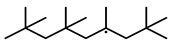	${\dot{C}}_{16}H_{32}OOH7\text{-}4$	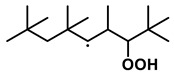
${\dot{C}}_{16}H_{33}\text{-}7$	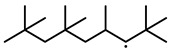	${\dot{C}}_{16}H_{32}OOH8\text{-}5$	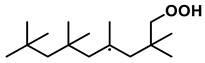
$C_{16}H_{33}\text{-}2{\dot{O}}_{2}$	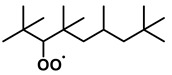	${\dot{C}}_{16}H_{32}OOH8\text{-}7$	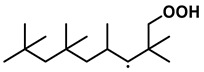
$C_{16}H_{33}\text{-}3{\dot{O}}_{2}$	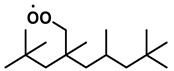	$C_{16}H_{32}OOH7\text{-}4{\dot{O}}_{2}$	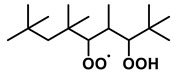
$C_{16}H_{33}\text{-}7{\dot{O}}_{2}$	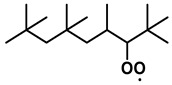	$C_{16}H_{32}OOH8\text{-}5{\dot{O}}_{2}$	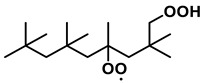
${\dot{C}}_{16}H_{32}OOH3\text{-}5$	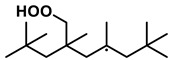		

**Table 3:** Updated rate constants for high-temperature reactions (P = Primary carbon, S = Secondary carbon, T = Tertiary carbon).

Reaction Class	Site	Modification	Source
Fuel decomposition		A × 2	Raza et al. [5]
Alkyl radical decomposition	P	A × 0.98	Raza et al. [5]
	S	A × 1.07	
	T	A × 0.69	

**Table 5:** Recommended values for the reaction rate constants of $\dot{R}$ + $O_{2}$. P = Primary carbon, S = Secondary carbon, T = Tertiary carbon. Rate constant in Arrhenius form (*k* = *AT^n^* exp(*Ea*/*RT*)); units are cm^3^, K, mol, s, and kJ.

Site	*A*	*n*	*Ea*	Source
P	5.491 × 10^16^	−1.63	199	Miyoshi [39]
S	2.789 × 10^14^	−0.816	−536	
T	7.802 × 10^11^	0.325	−417	

**Table 6:** Recommended values for the reaction rate constants of $\dot{Q}OOH$ + $O_{2}$. P = Primary carbon, S = Secondary carbon, T = Tertiary carbon. Rate constant in Arrhenius form (*k* = *AT^n^* exp(*Ea*/*RT*)); units are cm^3^, K, mol, s, and kJ.

Site	*A*	*n*	*Ea*	Source
P	2.746 × 10^16^	−1.63	199	Miyoshi [39]
S	1.394 × 10^14^	−0.816	−536	
T	3.901 × 10^11^	0.325	−417	

**Table 7:** Recommended values for the reaction rate constants of $R{\dot{O}}_{2}$ ⇌ $\dot{Q}OOH$ isomerization. P = Primary carbon, S = Secondary carbon, T = Tertiary carbon. Location refers to spatial relation between carbon sites. Rate constant in Arrhenius form (*k* = *AT^n^* exp(*Ea*/*RT*)); units are cm^3^, K, mol, s, and kJ.

COO	CH	Location	*A*	*n*	*Ea*	Source
P	T	β	5.773 × 10^9^	0.8	2.71 × 10^4^	Sharma et al. [42]
S	T	β	1.863 × 10^10^	0.6	2.73 × 10^4^	
T	P	β	1.256 × 10^9^	1.2	3.35 × 10^4^	
T	S	β	8.143 × 10^9^	0.7	3.10 × 10^4^	
P	P	γ	2.973 × 10^6^	1.6	2.10 × 10^4^	
P	S	γ	1.700 × 10^7^	1.3	1.82 × 10^4^	
S	P	γ	1.199 × 10^7^	1.4	2.08 × 10^4^	
S	S	γ	8.793 × 10^10^	0.2	1.85 × 10^4^	
P	T	δ	4.270 × 10^6^	1.2	1.38 × 10^4^	
T	P	δ	1.028 × 10^6^	1.5	2.00 × 10^4^	
P	P	ε	2.451 × 10^5^	1.5	1.99 × 10^4^	
P	S	ε	3.250 × 10^6^	1	1.82 × 10^4^	
S	P	ε	2.451 × 10^5^	1.5	1.99 × 10^4^	

**Table 8:** Recommended values for the reaction rate constants of $R{\dot{O}}_{2}$ = olefin + $H{\dot{O}}_{2}$ and ${\dot{O}}_{2}QOOH$ = EROOH + $H{\dot{O}}_{2}$. P = Primary carbon, S = Secondary carbon, T = Tertiary carbon. Rate constant in Arrhenius form (*k* = *AT^n^* exp(*Ea*/*RT*)); units are cm^3^, K, mol, s, and kJ.

COO	CH	*A*	*n*	*Ea*	Source
P	T	2.52 × 10^8^	1.32	2.79 × 10^4^	Villano et al. [41]
S	T	7.00 × 10^10^	0.71	3.01 × 10^4^	
T	P	4.08 × 10^9^	0.89	2.95 × 10^4^	
T	S	5.62 × 10^10^	0.58	2.96 × 10^4^	

**Table 9:** Modifications to the rate constants for the formation reactions of cyclic ethers.

Reactions	*A*	*A*_0_×	Source
${\dot{C}}_{16}H_{32}OOH1\text{-}1$ = $C_{16}H_{32}O1\text{-}1$ + $\dot{O}H$	5.3468 × 10^11^	1.73	Heng [49]
${\dot{C}}_{16}H_{32}OOH1\text{-}2$ = $C_{16}H_{32}O1\text{-}2$ + $\dot{O}H$	2.2668 × 10^12^		
${\dot{C}}_{16}H_{32}OOH3\text{-}2$ = $C_{16}H_{32}O2\text{-}3$ + $\dot{O}H$	2.1985 × 10^12^	1.68	
${\dot{C}}_{16}H_{32}OOH3\text{-}3$ = $C_{16}H_{32}O3\text{-}3$ + $\dot{O}H$	5.1857 × 10^11^		
${\dot{C}}_{16}H_{32}OOH3\text{-}4$ = $C_{16}H_{32}O3\text{-}4$ + $\dot{O}H$	2.1985 × 10^12^		
${\dot{C}}_{16}H_{32}OOH3\text{-}5$ = $C_{16}H_{32}O3\text{-}5$ + $\dot{O}H$	1.8628 × 10^10^		
${\dot{C}}_{16}H_{32}OOH5\text{-}2$ = $C_{16}H_{32}O2\text{-}5$ + $\dot{O}H$	5.0889 × 10^10^	1.79	
${\dot{C}}_{16}H_{32}OOH5\text{-}3$ = $C_{16}H_{32}O3\text{-}5$ + $\dot{O}H$	5.0889 × 10^10^		
${\dot{C}}_{16}H_{32}OOH5\text{-}8$ = $C_{16}H_{32}O5\text{-}8$ + $\dot{O}H$	5.0889 × 10^10^		
${\dot{C}}_{16}H_{32}OOH8\text{-}5$ = $C_{16}H_{32}O5\text{-}8$ + $\dot{O}H$	2.2191 × 10^10^	2.00	
${\dot{C}}_{16}H_{32}OOH8\text{-}7$ = $C_{16}H_{32}O7\text{-}8$ + $\dot{O}H$	2.6190 × 10^12^		
${\dot{C}}_{16}H_{32}OOH8\text{-}8$ = $C_{16}H_{32}O8\text{-}8$ + $\dot{O}H$	6.1776 × 10^11^		

**Table 10:** Recommended values for the reaction rate constants of ketohydroperoxide formation. P = Primary carbon, S = Secondary carbon, T = Tertiary carbon. Location refers to spatial relation between carbon sites. Rate constant in Arrhenius form (*k* = *AT^n^* exp(*Ea*/*RT*)); units are cm^3^, K, mol, s, and kJ.

COO	COOH	Location	*A*	*n*	*Ea*	Source
T	P	β	3.662 × 10^7^	1.6	2.79 × 10^4^	Sharma et al. [42]
T	S	β	5.242 × 10^8^	1.7	2.6 × 10^4^	
P	P	γ	5.188 × 10^4^	1.9	1.88 × 10^4^	
P	S	γ	1.157 × 10^2^	2.9	1.7 × 10^4^	
S	P	γ	5.188 × 10^4^	1.9	1.88 × 10^4^	
S	S	γ	3.508 × 10^2^	3.1	1.75 × 10^4^	
T	P	δ	1.884 × 10^3^	2.2	1.64 × 10^4^	
P	P	ε	1.115 × 10^3^	1.8	1.66 × 10^4^	
P	S	ε	3.990 × 10^3^	1.9	1.49 × 10^4^	

**Table 11:** Recommended values for the reaction rate constants of formation and decomposition of $\dot{P}{\left(OOH\right)}_{2}$. P = Primary carbon, S = Secondary carbon, T = Tertiary carbon. Location refers to spatial relation between carbon sites. Rate constant in Arrhenius form (*k* = *AT^n^* exp(*Ea*/*RT*)); units are cm^3^, K, mol, s, and kJ.

COO	CH	Location	*A*	*n*	*Ea*	Source
P	T	β	2.309 × 10^9^	0.8	2.71 × 10^4^	Sharma et al. [42]
S	T	β	7.449 × 10^9^	0.6	2.73 × 10^4^	
T	P	β	5.024 × 10^8^	1.2	3.35 × 10^4^	
T	S	β	3.257 × 10^9^	0.7	3.10 × 10^4^	
P	P	γ	1.189 × 10^6^	1.6	2.10 × 10^4^	
P	S	γ	6.798 × 10^6^	1.3	1.82 × 10^4^	
S	P	γ	4.795 × 10^6^	1.4	2.08 × 10^4^	
S	S	γ	3.517 × 10^10^	0.2	1.85 × 10^4^	
P	T	δ	1.708 × 10^6^	1.2	1.38 × 10^4^	
T	P	δ	4.111 × 10^5^	1.5	2.00 × 10^4^	
P	P	ε	9.803 × 10^4^	1.5	1.99 × 10^4^	
P	S	ε	1.300 × 10^6^	1	1.82 × 10^4^	
S	P	ε	9.803 × 10^4^	1.5	1.99 × 10^4^	

**Table 12:** Recommended values for the reaction rate constants of the decomposition of $\dot{P}{\left(OOH\right)}_{2}$. P = Primary carbon, S = Secondary carbon, T = Tertiary carbon. Location refers to spatial relation between carbon sites. Rate constant in Arrhenius form (*k* = *AT^n^* exp(*Ea*/*RT*)); units are cm^3^, K, mol, s, and kJ.

	COO	CH	*A*	*n*	*Ea*	Source
$\dot{P}{\left(OOH\right)}_{2}$ = olefins + $H{\dot{O}}_{2}$	P	T	2.52 × 10^8^	1.32	2.79 × 10^4^	Villano et al. [46]
	S	T	7.00 × 10^10^	0.71	3.01 × 10^4^	
	T	P	4.08 × 10^9^	0.89	2.95 × 10^4^	
	T	S	5.62 × 10^10^	0.58	2.96 × 10^4^	
$\dot{P}{\left(OOH\right)}_{2}$ $=\mathrm{olefins}+R=O+\dot{O}H$			3.081 × 10^13^	0	530(∆rxnH298) + 32,400	Villano et al. [46]
$\dot{P}{\left(OOH\right)}_{2}$ $=\mathrm{olefins}+\dot{Q}OOH$			1.392 × 10^14^	0	930(∆rxnH298) + 7200	Villano et al. [46]

## Data Availability

Data are contained within the article and Supplementary Materials.

## Supplementary Materials

The following supporting information can be downloaded at: https://www.mdpi.com/article/10.3390/molecules31091403/s1, File S1: HMN-MECH.txt and File S2: HMN‐THERM.txt.

## Abbreviation

$\dot{P}{\left(OOH\right)}_{2}$	Alkyl-dihydroperoxides
NTC	Negative temperature coefficient
ST	Shock tube
RCM	Rapid compression machine
JSR	Jet-stirred reactor
$R{\dot{O}}_{2}$	Alkyl peroxy radicals
$\dot{Q}OOH$	Hydroperoxyalkyl radicals
${\dot{O}}_{2}QOOH$	Peroxyhydroperoxyalkyl radicals
KHP	Ketohydroperoxides
GAVs	Group additivity values
IDT	Ignition delay time
PLOG	Pressure-dependent Logarithmic interpolation
TST	Transition state theory
NNN	Next nearest neighbor
P	Primary carbon
S	Secondary carbon
T	Tertiary carbon
*A*	Pre-exponential factor
*n*	Temperature exponent
*Ea*	Activation energy
VTST	Variational transition state theory
VRC-TST	Variable reaction coordinate transition state theory
CBS-QB3	Complete basis set-quadratic becke3
SFT	Scaled Frequency Treatment
